# USP8 maintains embryonic stem cell stemness via deubiquitination of EPG5

**DOI:** 10.1038/s41467-019-09430-4

**Published:** 2019-04-01

**Authors:** Haifeng Gu, Xingxing Shi, Chao Liu, Chaoqun Wang, Ning Sui, Yu Zhao, Jiaqi Gong, Fuping Wang, Hong Zhang, Wei Li, Tongbiao Zhao

**Affiliations:** 1 State Key Laboratory of Stem Cell and Reproductive Biology, Institute of Zoology, Chinese Academy of Sciences, Beijing, 100101 China; 2 University of Chinese Academy of Sciences, Beijing, 100049 China; 3 Qufu Normal University, Qufu, 273165 China; 4 Hebei University, Baoding, 071002 China; 5 Institute of Biophysics, Chinese Academy of Sciences, Beijing, 100101 China

## Abstract

Embryonic stem cells (ESCs) can propagate in an undifferentiated state indefinitely in culture and retain the potential to differentiate into any somatic lineage as well as germ cells. The catabolic process autophagy has been reported to be involved in ESC identity regulation, but the underlying mechanism is still largely unknown. Here we show that EPG5, a eukaryotic-specific autophagy regulator which mediates autophagosome/lysosome fusion, is highly expressed in ESCs and contributes to ESC identity maintenance. We identify that the deubiquitinating enzyme USP8 binds to the Coiled-coil domain of EPG5. Mechanistically, USP8 directly removes non-classical K63-linked ubiquitin chains from EPG5 at Lysine 252, leading to enhanced interaction between EPG5 and LC3. We propose that deubiquitination of EPG5 by USP8 guards the autophagic flux in ESCs to maintain their stemness. This work uncovers a novel crosstalk pathway between ubiquitination and autophagy through USP8-EPG5 interaction to regulate the stemness of ESCs.

## Abstract (web summary)

Autophagy is implicated in self-renewal of stem cells, including embryonic stem cells (ES cells). Here the authors demonstrate EPG5 is highly expressed in ES cells, and its deubiquitylation by USP8 stimulates its interaction with LC3 to promote autophagy and maintenance of stemness.

## Introduction

Autophagy is a highly conserved lysosome-mediated catabolic process in eukaryotic cells^1–3^. It was first defined as a bulk degradation process that generates resources to meet the cell’s requirements for metabolites and energy under stress conditions^4,5^. However, increasing numbers of studies have indicated that basal autophagy acts as a critical process to maintain cellular homeostasis by removing misfolded or aggregation-prone proteins and damaged organelles^6–8^.

Recently, significant progress was achieved in understanding the function of autophagy in stem cell regulation. In adult stem cells, increasing evidence suggests that autophagy is not only critical for enhancing the ability to resist stress conditions but is also essential for self-renewal and differentiation^9–13^. Adult stem cells, for example hematopoietic stem cells (HSCs), rely on basal autophagy to clear the active and healthy mitochondria, thereby keeping their metabolic rate low in order to maintain a quiescent pool^9^. In contrast, embryonic stem cells (ESCs) maintain a high autophagic flux to ensure a fast metabolic rate for rapid proliferation and self-renewal^14^. In addition, basal autophagy has been identified to degrade the mitochondria in mouse ESCs, thus maintaining mitochondrial homeostasis. In *Atg3*-knockout ESCs, dysfunctional autophagy inhibits mitochondrial removal, resulting in accumulation of the abnormal mitochondria and attenuated pluripotency^15^. These data suggest that high autophagic flux is an ESC-intrinsic characteristic and plays critical roles in ESC self-renewal and pluripotency. Thus, the molecules that promote autophagy may be beneficial for the maintenance of ESC pluripotency. To this end, we perform a screen for molecules that promote autophagy in mouse ESCs and identify EPG5, a metazoan-specific autophagy regulator, as a new guardian of ESC stemness. Moreover, using the coiled-coil domain of EPG5 as a bait, we define a novel EPG5-interacting protein, USP8, which is highly expressed in ESCs and guards ESC identity through deubiquitination of EPG5.

## Results

### EPG5 is essential for ESC self-renewal and pluripotency

**Fig. 1:**
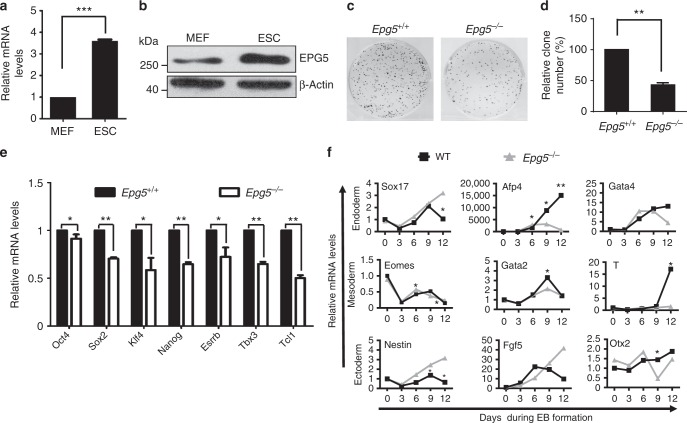
EPG5 maintains ESC self-renewal and pluripotency. **a** The mRNA expression of *Epg5* in ESCs and MEFs. Error bars indicate the standard deviation (SD) (*n* = 3). Three independent biological replicates. ***P < 0.001 by unpaired two-tailed Student’s *t* test. **b** Western blot analysis of whole-cell extracts from MEFs and ESCs. β-Actin served as a loading control. Images are representative of three independent experiments. **c**, **d** Colony-formation assay of *Epg5*^+/+^ and *Epg5*^*−*/*−*^ ESCs. Colonies formed by ESCs were stained with alkaline phosphatase (AP). Error bars indicate the SD (*n* = 5). Five independent biological replicates. **P < 0.01 by unpaired two-tailed Student’s *t* test. **e** ESC pluripotency is impaired by *Epg5* depletion. The relative mRNA expression of pluripotency genes in *Epg5*^+/+^ and *Epg5*^*−*/*−*^ ESCs was detected by quantitative PCR. Error bars indicate the SD (*n* = 3). Three independent biological replicates. **P < 0.01, *P < 0.05 by unpaired two-tailed Student’s *t* test. **f** Absence of EPG5 impairs ESC lineage specification. The relative mRNA expression levels of genes representative of the ectoderm, mesoderm, and endoderm were detected during embryonic body (EB) differentiation by quantitative PCR on the indicated days. Data shown are representative of three independent experiments. Error bars indicate the SD (*n* = 4). Four independent biological replicates. **P < 0.01, *P < 0.05 by two-way ANOVA with Bonferroni’s correction (WT compared with *Epg5*^*−*/*−*^)

We first screened the expression of a panel of autophagy-regulatory genes in somatic cells and ESCs using existing transcriptome data as previously described, and found that EPG5 is highly expressed in ESCs^14,16^. EPG5, a metazoan-specific autophagy protein, regulates autophagy by promoting fusion of autophagosomes with lysosomes and/or late endosomes^17–19^. Using quantitative PCR and western blot assays, we confirmed that *Epg5* is highly expressed in mouse ESCs at both the mRNA and protein levels compared with mouse embryonic fibroblasts (MEFs) (Fig. 1a, b). In addition, we detected that *Epg5* is highly expressed in iPSC in comparation with mouse tail fibroblast (TIF) and neuron stem cells (NSC) (Supplementary Figure 6c). The expression of *Epg5* is gradually decreased upon embryoid body differentiation (Supplementary Figure 6d). The *Epg5* expression in human pluripotent stem cells like ESC and iPSC is higher than that of human somatic cells as well (Supplementary Figure 10a). To investigate whether EPG5 is involved in the regulation of ESC identity, we designed specific small interfering RNAs (siRNAs) targeting *Epg5* and found that transient inhibition of *Epg5* leads to ESC differentiation and reduced expression of pluripotency genes in both mouse and human ESCs (Supplementary Figure 1a–d, Supplementary Figure 10b, 10c).

We then designed an sgRNA targeting the first exon of the mouse *Epg5* gene and knocked out *Epg5* in ESCs using the CRISPR-Cas9 system (Supplementary Figure 1e). Western blotting confirmed the absence of EPG5 protein expression in *Epg5*-knockout (*Epg5*^*−/−*^) ESCs (Supplementary Figure 1f). Furthermore, the *Epg5*^*−/−*^ ESCs have a normal karyotype (Supplementary Figure 1g). Using colony-formation assays, we found that depletion of *Epg5* in ESCs significantly inhibited the colony-formation efficiency compared with wild-type (WT) ESCs (Fig. 1c, d). *Epg5* deletion did not affect ESC apoptosis and expression of differentiation marker genes (Supplementary Figure 2a, b and Supplementary Figure 9). These results indicated that EPG5 is essential for ESC self-renewal.

To test whether loss of EPG5 affects ESC pluripotency, we assessed the mRNA expression of pluripotency genes by quantitative PCR. We found that pluripotency gene expression was reduced in *Epg5*^*−/−*^ versus *Epg5*^*+/+*^ ESCs, suggesting that depletion of *Epg5* leads to compromised pluripotency in ESCs (Fig. 1e). In support of this hypothesis, *Epg5*^*−/−*^ ESCs showed abnormal embryonic body (EB) differentiation, characterized by delayed expression of certain mesodermal marker genes (Fig. 1f). Together, these data suggest that EPG5 is a critical regulator of ESC pluripotency.

### The coiled-coil domain of EPG5 regulates ESC identity

**Fig. 2:**
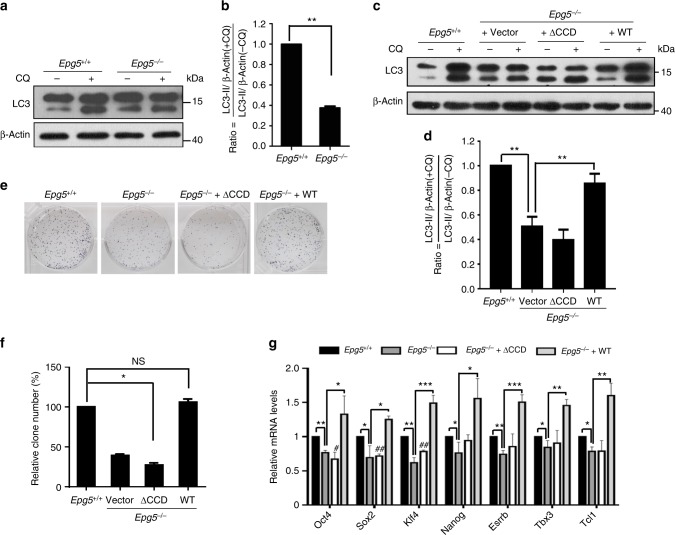
EPG5 depends on its coiled-coil domain to regulate ESC identity. **a** Western blotting for LC3 and β-actin in *Epg5*^+/+^ and *Epg5*^*−*/*−*^ ESCs. β-Actin served as a loading control. Samples were treated with or without the autophagy inhibitor chloroquine (50 μM) for 2 h. Images are representative of three independent experiments. **b** Quantification of autophagic flux in **a** by calculating the ratio of LC3-II with or without CQ treatment. Error bars indicate the SD (*n* = 3). Three independent biological replicates. **P < 0.01 by unpaired two-tailed Student’s *t* test. **c** Introduction of wild-type *Epg5* but not an *Epg5* mutant lacking the coiled-coil domain (∆CCD) into *Epg5*^*−*/*−*^ cells ameliorates the impaired autophagic flux. **d** Quantification of the autophagic flux by calculating the ratio of LC3-II with or without CQ treatment in **c**. Error bars indicate the SD (*n* = 3). Three independent biological replicates. **P < 0.01 by unpaired two-tailed Student’s *t* test. **e**, **f** Introduction of WT *Epg5* but not the ∆CCD *Epg5* mutant into *Epg5*^*−*/*−*^ cells restored the colony-formation ability. ESC colonies were stained with AP. Error bars indicate the SD (*n* = 3). Three independent biological replicates. NS not significant. *P < 0.05 by unpaired two-tailed Student’s *t* test. **g** Reduced pluripotency gene expression in *Epg5*^*−*/*−*^ ESCs is rescued by introduction of WT *Epg5* but not the ∆CCD *Epg5* mutant. Error bars indicate the SD (*n* = 3). Three independent biological replicates. *P < 0.05, **P < 0.01, ***P < 0.001, ^#^*P* < 0.05, ^##^P < 0.01 by unpaired two-tailed Student’s t test. # (Epg5^-/-^ +∆CCD compared with Epg5^+/+^)

To investigate how EPG5 regulates ESC pluripotency, we first determined whether EPG5 regulates autophagic flux, since high autophagic flux has been shown to maintain ESC self-renewal and pluripotency^14^. We detected the autophagic flux by measuring LC3-II turnover or degradation of EGFP-LC3 or colocalization of LC3 with LAMP1 in cells that were either treated or untreated with the autophagy inhibitor chloroquine. The results showed that depletion of EPG5 significantly decreased autophagic flux in ESCs (Fig. 2a, b; Supplementary Figure 8a–e). We then performed bioinformatic analysis and found that EPG5 has a coiled-coil domain, which is a highly versatile protein-folding motif that can act as a recognition system for cellular signal transduction and provide mechanical stability to cells, as well as carrying out other important functional tasks^20–22^. To investigate whether the coiled-coil domain of EPG5 regulates autophagic flux and thus ESC identity, gain-of-function assays were performed by introducing either WT *Epg5* or an *Epg5* mutant lacking the coiled-coil domain (∆CCD) into *Epg5*^*−/−*^ ESCs (Supplementary Figure 6a). We found that expressing WT *Epg5* but not the ∆CCD *Epg5* mutant in *Epg5*^*−/−*^ ESCs restored the decreased autophagic flux and ameliorated the defective self-renewal and pluripotency (Fig. 2c–g). Taken together, these data provide evidence that the coiled-coil domain of EPG5 is critical for ESC identity maintenance.

### EPG5 directly interacts with USP8

**Fig. 3:**
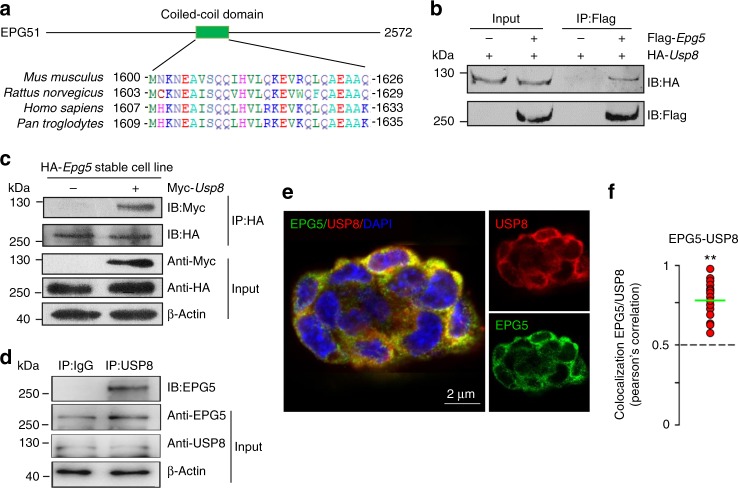
EPG5 directly interacts with USP8 through its coiled-coil motif. **a** Sequence alignment of the EPG5 coiled-coil domain in mouse, rat, human, and chimpanzee. **b** USP8 interacts with EPG5 in HEK293T cells transfected with HA-USP8 and/or Flag-EPG5. **c** EPG5 interacts with USP8 in stable ESC lines harboring HA-EPG5 and/or Myc-USP8 vectors. **d** Endogenous EPG5 is coimmunoprecipitated with USP8 in ESCS. **e** Confocal fluorescence microscopy images showing that endogenous EPG5 (green) co-localizes with USP8 (red) in ESCs. DAPI (blue). **f** Pearson’s correlation illustrated EPG5-USP8 colocalization. Red plots show colocalization values for individual cells, for >20 cells per condition. In all cases, similar results were seen in three independent experiments. Scale bars: 2 μm. **P < 0.01 by unpaired two-tailed Student’s *t* test in **f**

The coiled-coil domain of EPG5 is conserved in mammals (Fig. 3a). To investigate the molecular mechanisms that regulate EPG5 and thus the pluripotency of ESCs, we synthesized a peptide corresponding to the coiled-coil motif of mouse EPG5 and used it as a bait to trap EPG5-interacting proteins. LC-MS/MS analysis identified nine coiled-coil domain-interacting proteins with more than 13 peptide reads (Supplementary Figure 3a, b). One of the nine identified proteins is USP8, a deubiquitinating enzyme previously implicated in mitochondrial quality control, endosomal trafficking, and cellular proliferation^23–26^. To examine whether USP8 binds to EPG5 in cells, we transfected Flag-tagged *Epg5* and/or HA-tagged *Usp8* in HEK293T cells and performed coimmunoprecipitation (co-IP) assays. The results showed that USP8 is immunoprecipitated by EPG5, indicating that there is an interaction between EPG5 and USP8 (Fig. 3b). To further confirm that EPG5 interacts with USP8 in ESCs, stable ESC lines harboring HA-*Epg5* or Myc-*Usp8* vectors were established. Co-IP assay confirmed the interaction between EPG5 and USP8 in ESCs (Fig. 3c). Then, by using anti-USP8 antibody, we found that endogenous EPG5 can be immunoprecipitated by USP8 as well in ESCs (Fig. 3d). Furthermore, immunofluorescence microscopy showed that EPG5 did directly interact with USP8 in ESCs (Fig. 3e, f). These data support that EPG5 binds to USP8.

### USP8 is required for regulating ESC identity

**Fig. 4:**
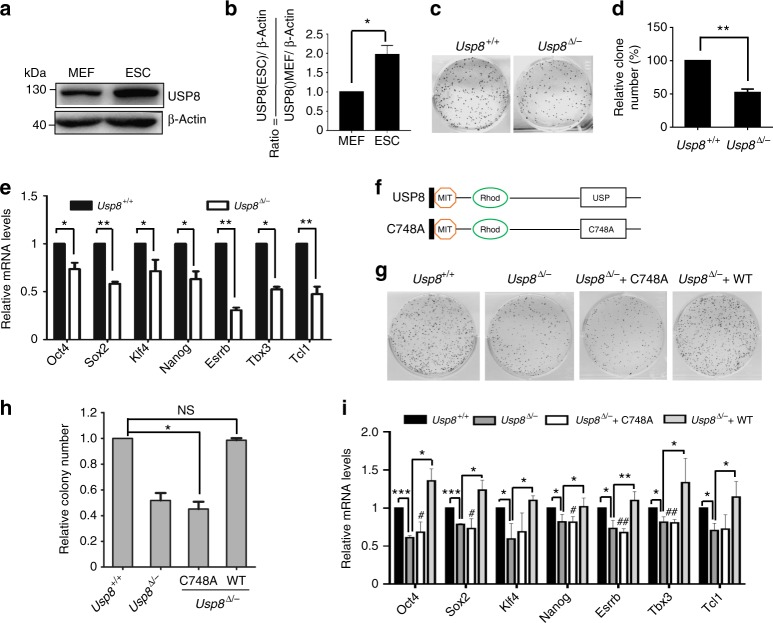
The deubiquitin activity of USP8 is required for ESC identity maintenance. **a**,**b** Western blot analysis of whole-cell extracts from MEFs and ESCs using anti-USP8 antibody. β-Actin served as a loading control. Images are representative of three independent experiments. Error bars indicate the SD (*n* = 3). Three independent biological replicates. *P < 0.05 by unpaired two-tailed Student’s *t* test. **c**, **d** Colony-formation assay of *Usp8*^+/+^ and *Usp8*^▵/*−*^ ESCs. ESC colonies were stained by AP. Error bars indicate the SD (*n* = 3). Three independent biological replicates. **P < 0.01 by unpaired two-tailed Student’s *t* test. **e** ESC pluripotency is impaired by decrease of *Usp8*. The relative mRNA expression of pluripotency genes in *Usp8*^+/+^ and *Usp8*^▵/*−*^ ESCs was assayed by quantitative PCR. Error bars indicate the SD (*n* = 3). Three independent biological replicates. *P < 0.05; **P < 0.01 by unpaired two-tailed Student’s *t* test. **f** Schematic illustration of the domain organization of USP8 and the location of the C748A mutation. Boundaries of individual domains and the amino acid numbering of the catalytic Cys residue are indicated according to mouse USP8. USP, ubiquitin-specific protease domain with catalytic Cys 748. MIT-Rhod (amino acid residues 1–319). **g**,**h** The reduced colony formation ability of *Usp8*^▵/*−*^ ESCs is rescued by introduction of WT *Usp8*, but not the C748A *Usp8* mutant. Error bars indicate the SD (*n* = 3). Three independent biological replicates. NS not significant. *P < 0.05 by unpaired two-tailed Student’s *t* test. **i** Introduction of WT but not the C748A *Usp8* mutant into *Usp8*^▵/*−*^ cells restores the expression of pluripotency genes. The relative mRNA expression of pluripotency genes was determined by quantitative PCR. Error bars indicate the SD (*n* = 3). Three independent biological replicates. NS not significant. *P < 0.05, **P < 0.01, ***P < 0.001, ^#^P < 0.05, ^##^P < 0.01 by unpaired two-tailed Student’s t test. # (Usp8^∆/-^ +∆C748A compared with Usp8^+/+^)

We next asked whether USP8 functions in ESCs. We performed western blot assays and found that USP8 is highly expressed in both mouse and human pluripotent stem cells (including ESCs and induced pluripotent stem cells) compared with somatic cells like mouse embryonic fibroblast (MEF), TIF, neuron stem cell (NSC), and human fibroblast (HEF), foreskin (FS) cells, respectively (Fig. 4a, b; Supplementary Figures 6c, 10a). We then performed RNAi assays using siRNAs targeting both mouse and human *Usp8* gene. We found that decreasing the expression of *Usp8* leads to ESC differentiation and lower expression of pluripotency genes (Supplementary Figures 3c–e,  10b, 10d). To further investigate how USP8 affects ESC identity, we aimed to generate *Usp8-*knockout ESC lines by CRISPR-Cas9-mediated gene targeting (Supplementary Figure 3f). Interestingly, we did not obtain *Usp8* double-knockout ES lines within the 60 positive lines harboring indels, indicating that loss of total *Usp8* may be lethal (Supplementary Figure 3g). Then we used an ES line, which has a 12 base deletion in one allele and a 13 base deletion in another allele (*Usp8*^▵/*−*^), for further analysis. These *Usp8*^▵/*−*^ ESCs have a decreased USP8 protein expression and a normal karyotype (Supplementary Figures 3h and 6b). Colony-formation assays were then employed to investigate the effects of USP8 on ESC self-renewal. In contrast to the WT ESCs, *Usp8*^▵/*−*^ ESCs formed a significantly lower number of colonies (Fig. 4c, d). Decreasing USP8 did not affect ESC apoptosis (Supplementary Figure 4a, 4b). These data indicate that USP8 is critical for ESC self-renewal. Furthermore, the reduced expression of pluripotent genes in *Usp8*^▵/*−*^ versus WT ESCs suggests the involvement of USP8 in pluripotency regulation in ESCs (Fig. 4e). Taken together, these data indicated that USP8 is important for maintenance of ESC identity.

Previous studies reported that the C748A mutant of USP8 had markedly reduced deubiquitinase activity and failed to deubiquitinate the substrates of USP8^24^. To investigate whether the aberrant self-renewal and pluripotency of *Usp8*^▵/*−*^ ESCs were directly caused by the decrease of USP8 deubiquitinating activity, gain-of-function assays were performed by introducing a *Usp8* expression constructs into *Usp8*^▵/*−*^ ESCs. We established stable *Usp8*^▵/*−*^ ESC lines carrying WT *Usp8* or the deubiquitinase-dead mutant of *Usp8* (C748A) (Fig. 4f). The colony-forming ability of *Usp8*^▵/*−*^ ESCs was rescued in stable *Usp8*^▵/*−*^ ESC lines carrying WT *Usp8*, but not the C748A mutant *Usp8* (Fig. 4g, h). Similarly, the decreased expression of pluripotent genes in *Usp8*^▵/*−*^ ESC lines was restored by WT *Usp8*, but not the C748A mutant (Fig. 4i). Together, these data suggested that USP8 depends on its deubiquitinase activity to regulate the self-renewal and pluripotency of ESCs.

### USP8 deubiquitinates EPG5 by removing K63-ubiquitin chains

**Fig. 5:**
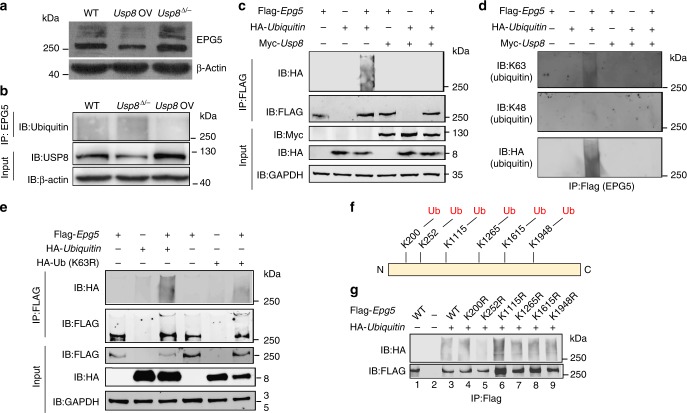
USP8 deubiquitinates the EPG5. **a** Western blot analysis of whole-cell extracts from WT, *Usp8*-overexpression and *Usp8*^▵/*−*^ ESC lines using anti-EPG5 antibody. β-Actin served as a loading control. Images are representative of three independent experiments. **b** Lysates from WT, *Usp8*-overexpression and *Usp8*^▵/*−*^ ESC lines were immunoprecipitated with anti-EPG5 and immunoblotted with anti-ubiquitin. Input cell lysates were immunoblotted with anti-USP8 and β-actin as controls. **c** Lysates of HEK293T cells transfected with plasmids encoding Flag-*Epg5*, HA-*Ubiquitin* and Myc-*Usp8* were immunoprecipitated with anti-Flag and immunoblotted with anti-HA and anti-FLAG. Input cell lysates were immunoblotted with anti-Myc, anti-HA, and anti-GAPDH as controls. **d** Lysates of HEK293T cells transfected with plasmids encoding Flag-*Epg5*, HA-*Ubiquitin*, and Myc-*Usp8* were immunoprecipitated with anti-Flag and immunoblotted with anti-K63, anti-K48, and anti-HA antibodies. **e** Lysates of HEK293T cells transfected with plasmids encoding Flag-*Epg5*, HA-*Ubiquitin*, or HA-*Ubiquitin* (K63R) were immunoprecipitated with anti-Flag and immunoblotted with anti-HA and anti-FLAG antibodies. Input cell lysates were immunoblotted with anti-FLAG, anti-HA and anti-GAPDH antibodies as controls. **f** Proteins from ESCs expressing Flag-*Epg5* were analyzed by LC-MS/MS mass spectrometry. Ubiquitination sites identified on EPG5 are indicated. **g**)Immunoblot analysis of protein extracts of HEK293T cells transfected with HA-*Ubiquitin* together with plasmids expressing WT Flag-*Epg5* or K200R, K252R, K1115R, K1265R, K1615R, and K1948R mutants of Flag-*Epg5*

To test whether USP8 deubiquitinates EPG5, we examined the ubiquitination levels in either *Usp8*-overexpression or *Usp8*^▵/*−*^ ESCs. When we overexpressed *Usp8* in ESCs, the ubiquitin modification of EPG5 decreased, while reduced *Usp8* expression increased EPG5 ubiquitination (Fig. 5a, b). These data indicate that USP8 deubiquitinates EPG5. To further confirm this hypothesis, we transfected Flag-*Epg5*, HA-*Ubiquitin*, and Myc-*Usp8* or Myc-*Usp8* (C748A) into HEK293T cells and performed coimmunoprecipitation assays. The results showed that the extensive ubiquitination of EPG5 was completely abolished by overexpressing WT, but not C748A mutant *Usp8* (Fig. 5c; Supplementary Figure 5a). These data suggested that USP8 directly deubiquitinates EPG5.

We next determined which kind of ubiquitin modification is removed by USP8. Ubiquitinated EPG5 was detected by an anti-K63, but not an anti-K48 ubiquitin antibody in HEK293T cells transfected with *Epg5* and *Ubiquitin*, which indicates that EPG5 is conjugated with K63-ubiquitin chains (Fig. 5d). Mutating K63 of ubiquitin to R63 dramatically decreased the ubiquitination of EPG5 (Fig. 5e). Furthermore, the K63-ubiquitination of EPG5 was efficiently removed by WT, but not C748A mutant USP8 (Fig. 5d; Supplementary Figure 5a). These data indicate that EPG5 undergoes K63-linked ubiquitination and is specifically deubiquitinated by USP8.

We then purified the EPG5 protein by immunoprecipitation using anti-FLAG antibody in HEK293T cells expressing Flag-*Epg5*. LC-MS/MS analysis of the immunoprecipitated proteins identified six lysines in EPG5 that are potentially conjugated to ubiquitin (Fig. 5f). We mutated each of the six lysines and transfected the mutant EPG5 proteins with HA-*Ubiquitin*. Co-IP assays showed that mutation of lysine 252, but not the lysine at other positions, eliminated EPG5 ubiquitination (Fig. 5g). This indicates that EPG5 is conjugated to K63-ubiquitin chains via K252.

### USP8 regulates ESC identity through deubiquitinating EPG5

To test whether USP8 regulates ESC identity through EPG5 deubiquitination, we performed rescue assay by overexpression WT, △LC3, or K252R mutant EPG5 in si*Usp8* ESCs (Supplementary Figure 7a, b). Interestingly, we found only K252R mutant EPG5, but not WT or △LC3 mutant EPG5, could compensate the defective colony formation and pluripotent gene expression lead by USP8 deterioration (Supplementary Figure 7c–e). These data support that USP8 regulates ESC self-renewal and pluripotency through deubiquitination of EPG5 at K252.

### USP8 regulates the interaction between EPG5 and LC3

**Fig. 6:**
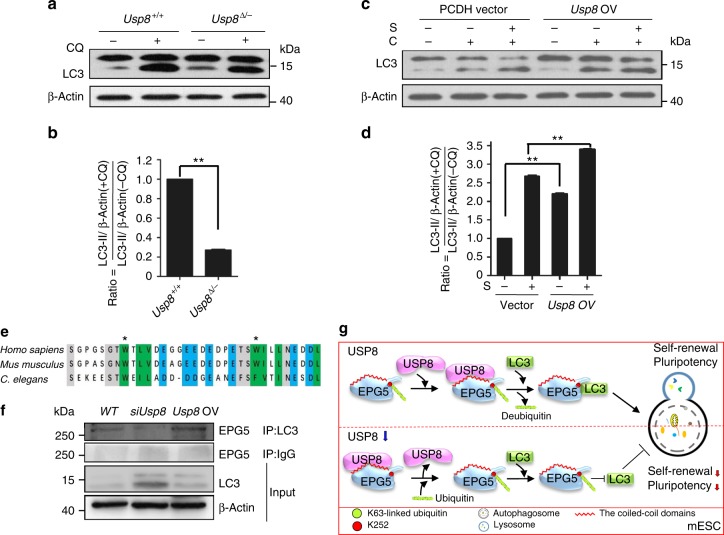
USP8 dynamically regulates the EPG5-LC3 interaction. **a** Western blotting for LC3 and β-actin in *Usp8*^+/+^ and *Usp8*^▵/*−*^ ESCs. β-Actin served as the loading control. Cells were treated with 50 μM chloroquine (CQ) for 2 h. **b** Quantification of autophagic flux in **a** by calculating the ratio of LC3-II with or without CQ treatment. Error bars indicate the SD (*n* = 3). Three independent biological replicates. **P < 0.01 by unpaired two-tailed Student’s *t* test. **c** Western blotting for LC3 and β-actin in WT and *Usp8*-overexpression stable ESCs upon nutrient deprivation. β-Actin served as the loading control. Cells were treated with 50 μM chloroquine (C) for 2 h and/or starved (S) in EBSS for 2 h. **d** Quantification of autophagic flux by calculating the ratio of LC3-II with or without CQ treatment in **c**. Error bars indicate the SD (*n* = 3). Three independent biological replicates. **P < 0.01 by unpaired two-tailed Student’s *t* test. **e** Sequence alignment of the LC3-binding region (LIR) in human, mouse, and C. elegans EPG5 proteins. The aromatic residues in the two LIR motifs are indicated by asterisks. Conserved hydrophilic residues are in gray, conserved hydrophobic residues are in green, and conserved negatively charged residues are in blue. **f** Lysates of *Usp8*-overexpression and si*Usp8-knockdown* ESCs were immunoprecipitated with anti-LC3 or anti-IgG and immunoblotted with anti-EPG5 and anti-IgG antibodies. Input cell lysates were immunoblotted with anti-LC3 and anti-actin antibodies as controls. Cells were starved for 2 h before analysis. **g** A proposed model of the role of the USP8-EPG5-LC3 pathway in regulating ESC stemness

To test how USP8 regulates stemness through EPG5, we explored whether USP8 regulates autophagic flux. In contrast to WT ESCs, *Usp8*^▵/*−*^ ESCs showed significantly lower autophagic flux, while *Usp8*-overexpression ESCs had higher autophagic flux under both normal and starvation conditions (Fig. 6a–d; Supplementary Figure 8f, g). These data indicate that USP8 guards autophagic flux in ESCs.

EPG5 mediates autophagy by direct binding to LC3 using its LIR motif^19^, which is conserved in eukaryotes (Fig. 6e). Since USP8 directly deubiquitinates EPG5 at K252, we next investigated whether deubiquitination of EPG5 by USP8 impairs the interactions between EPG5 and LC3 and eventually affects ESC stemness. We performed coimmunoprecipitation assays using an anti-LC3 antibody in either *Usp8*-overexpression or *Usp8*^▵/*−*^ ESC lines. The results showed that overexpression of *Usp8* enhanced the interaction between LC3 and EPG5. In contrast, inhibition of *Usp8* abolished the interaction between LC3 and EPG5 (Fig. 6f). These data support that USP8 guards autophagic flux for ESC identity maintenance by reinforcing the interaction between EPG5 and LC3.

## Discussion

Previous studies demonstrated that autophagy contributes to somatic cell reprogramming^15,27–29^. These studies provided evidence to show that both canonical and noncanonical autophagy are executors of mitochondrial clearance during reprogramming, and are essential for acquisition of pluripotency. Recently, we determined that high autophagic flux is an intrinsic characteristic of mouse ESCs, and regulates the homeostasis of cellular organelles and proteins to maintain pluripotency^14,15^. Moreover, enhanced proteasome activity has been identified in human ESCs, and has been proposed to maintain an intact proteome for either self-renewal or generation of a specific cell lineage^30^. These findings strongly support the proposal that both autophagy and ubiquitin-mediated degradation pathways are critical catabolic processes in ESCs for regulation of cellular homeostasis and maintenance of ESC identity. Here, we defined a novel crosstalk pathway that links the two catabolic processes of autophagy and ubiquitination in ESCs to maintain ESC pluripotency. We revealed that the deubiquitinase USP8 maintains ESC identity by directly deubiquitinating EPG5 to consolidate the interaction between EPG5 and LC3 and sustain the normal autophagic flux for stemness maintenance (Fig. 6g).

At first, autophagy was believed to be a nonselective catabolic process; however, many lines of evidence revealed that autophagy mediates selective degradation of cellular protein aggregates and damaged organelles in conjunction with the ubiquitin modification system. Ubiquitination is a posttranslational modification process, in which ubiquitin is covalently linked to one or more lysine (K) of a substrate protein^31^. A recent study provides evidence that ubiquitin modification is a positive initiation signal for selective autophagy^8^. The autophagic adaptor protein p62 uses its ubiquitin-binding domain (UBA) and its LC3-interacting motif (LIR) to transfer this degradation signal to the lysosome^32,33^. In contrast, we show here that deubiquitination serves as a novel positive signal for maintenance of autophagic flux, and thus ESC identity. Consistent with this finding, a study has shown that the deubiquitinase USP19 stabilizes Beclin-1 by removing the K11-linked ubiquitin chains from Beclin-1, and positively regulates autophagy^34^. In addition, USP8-mediated removal of K6-linked Ub from Parkin has been shown to be required for efficient recruitment of Parkin to damaged mitochondria and subsequent mitophagy^26^.

The K48-linked ubiquitination is generally considered as a canonical signal to target proteins for proteasomal degradation, while K63-linked ubiquitin modification is usually thought to be a signal for non-proteolytic pathways^31,33,35^. Here, we identified K63-linked ubiquitin in EPG5 was removed by USP8, indicating EPG5 is not degraded through the ubiquitination-mediated proteasomal signaling pathway. In consistent with this presumption, EPG5 is found to be degraded under nutrient deprivation, and lysosome inhibitor chloroquine but not proteinase inhibitor MG132 could stabilize EPG5 when protein synthesis is blocked (Supplementary Figure 5b, c). These data suggested EPG5 is degraded through autophagy in ESCs.

In conclusion, we defined a novel mechanism, involving USP8 deubiquitination of EPG5, which underlies autophagy regulation in ESCs. We revealed that deubiquitination, like the reverse process of ubiquitination, can serve as a positive regulator of autophagy. Our findings thus extend our understanding of the crosstalk mechanisms and functions between autophagy and ubiquitin-mediated degradation.

## Methods

### Cell culture and vector construction

Human embryonic kidney 293T cells (HEK293T, ATCC, CRL-3216), human fibroblast (HEF^36^), human foreskin (FS, ATCC, PCS-200–010™) and mouse embryonic fibroblast (MEF^15^), TIF^15^ cells, and neural stem cell (NSC^14^) were cultured in the Dulbecco’s modified Eagle’s medium (DMEM) supplemented with 10% heat-inactivated fetal bovine serum, 2 mM glutamine, 1 mM sodium pyruvate, 100 μg/ml streptomycin, 100 U/ml penicillin, and 0.055 mM β-mercaptoethanol at 37 °C in 5% CO_2_. Both mouse ESCs^14^ and iPSCs^14^ were routinely maintained in the ESC medium (knockout DMEM with 15% heat-inactivated fetal bovine serum, 2 mM glutamine, 1 mM sodium pyruvate, 0.1 mM nonessential amino acids, 100 μg/ml streptomycin, 100 U/ml penicillin, 0.055 mM β-mercaptoethanol, and 1000 U/ml leukemia inhibitory factor). Both human ESCs^36^ and iPSCs^36^ were routinely maintained in the ESC medium (knockout DMEM, 2 mM glutamine (Invitrogen), 0.1 mM NEAA, 0.1 mM β-mercaptoethanol, 100 U/ml penicillin, 100 µg/ml streptomycin, and 20% knockout serum replacement). Mouse cDNA of *Epg5* and *Usp8* was cloned into the pCDH-CAG-mCherry lentivirus-vector. The HA, Flag or c-Myc tag was fused to the N terminus of *Epg5* or *Usp8* using standard cloning methods. All constructs generated were confirmed by DNA sequencing.

All animal experiments were approved by the Ethics Committee in the Institute of Zoology, Chinese Academy of Sciences in accordance with the Guidelines for Care and Use of Laboratory Animals established by the Beijing Association for Laboratory Animal Science.

### Virus preparation and viral infection

To prepare lentivirus for the knockdown experiments, HEK293T cells were transduced with the pSicoR vector and the lentivirus packaging vectors PSPAX2 and pMD2G using the calcium phosphate–DNA coprecipitation method. To prepare lentivirus for protein expression, HEK293T cells were transduced with pCDH-CAG-MCS-IRES-mCherry vectors and the packaging vectors PSPAX2 and pMD2G. Medium containing the virus was collected 48 h after transfection. The ES cells were infected in the collected viral supernatant in the presence of polybrene (8 µg/ml).

### siRNA design

Mouse target sequences were as follows: *Epg5* siRNA1: 5′-GGGACUGGUUAUUGAAUAA-3′, *Epg5* siRNA2: 5′-GAGGCUCUUUAAUGUAUAU-3′, *Usp8* siRNA1: 5′-GCGGGAUUCUCUAAAGAAA-3′, scramble: 5′-CUUACGCUGAGUACUUCGAAATT-3′. Human target sequences were as follows: *hEpg5* siRNA: 5′-ATTGCAAGCCACCCAATTTATTT-3′, *hUsp8* siRNA: 5′- CCACAGATTGATCGTACTAAATT-3′, *hOct4* siRNA: 5′-AAGGAUGUGGUCCGAGUGUGG-3′, scramble: 5′-CUUACGCUGAGUACUUCGAAATT-3′.

### *Epg5-*knockout and *Usp8*^▵/*−*^ ESCs

The sequences for the sgRNA targeting *Epg5* were as follows: F: CACCGTGGACCGACAGCGTGCCGAA; R: AAACTTCGGCACGCTGTCGGTTCCAC. The sequences for the *Usp8* sgRNA were as follows: F: CACCGTAACTTCGGTCTTCTTATTG; R: AAACCAATAAGAAGACCGAAGTTAC. After annealing, the oligos were ligated into the linearized vector px330. ESCs were transfected with this plasmid by electroporation. About 72 h after electroporation, single colonies were picked and sequenced. A region around the cutting site was amplified using the following primers: *Epg5*-F, 5′-AACTGCCTTTGTATCTTTGG-3′ Epg5-R, 5′-ACTGTCTCCTGGAACTCGTG-3′. *Usp8*-F, 5′-TATTTTTCAAGGTGTTTGTCCAGTT-3′ *Usp8*-R, 5′-ATTCTAATTCCAACCAATGGTATCC-3′. PCR products were first digested with T7 enzyme and then sequenced to identify the knockout ESCs.

### Reagents and antibodies

The following antibodies were used: mouse anti-ubiquitin (Santa Cruz(P4D1), sc-8017, 1:1000); rabbit anti-LC3 (Cell Signaling Technology (D11), #3868, 1:1000); mouse anti-LAMP1 (Cell Signaling Technology (D4O1S), #15665, 1:200); mouse anti–USP8 (Sigma-Aldrich (US872), SAB4200527, 1:1000); rabbit anti-EPG5 (Beiijng TDY Biotech CO., Ltd, TDY349F, 1:1000); mouse anti-actin (Sigma-Aldrich (AC-15), A5441, 1:5000); rabbit anti-HA tag (Abcam, ab9110, 1:1000); mouse anti-FLAG tag (Abmart, M20008, 1:1000); rabbit anti-Myc tag (Abmart, M20002, 1:1000); Alexa Fluor^®^ 488 donkey anti-rabbit IgG (H + L) (Invitrogen Thermo Fisher Scientific, A21206, 1:500); Alexa Fluor^®^ 555 donkey anti-mouse IgG (H + L) (Invitrogen Thermo Fisher Scientific, A21203,1: 200); Alexa Fluor^®^ 647 donkey anti-mouse IgG (H + L) (Invitrogen Thermo Fisher Scientific, A21235, 1:500). Chloroquine, Hoechst 33342, Baf-A1, and MG132 were obtained from Sigma-Aldrich.

### Colony-formation assay and AP staining

ESCs were seeded at 1000 cells/well in a six-well plate, and cultured for about 7 days. The colonies were stained using an Alkaline Phosphatase Assay Kit (Beyotime), and the colony numbers were analyzed by Image-Pro Plus software.

### Quantitative real-time PCR

Total RNA was extracted from MEFs, ESCs, and EBs with a total RNA isolation kit (GeneMark). The total RNA (2 µg) was reverse transcribed into cDNA using a SuperScript™ III First-Strand Synthesis System (Invitrogen). The primers used were as reported previously^15^ and listed in Supplementary Table 1.

### Immunoblotting

Cells were lysed in lysis buffer (50 mM Tris-HCl [pH 7.4], 150 mM NaCl, 1% NP-40, 0.5% sodium deoxycholate, 0.1% SDS and protease inhibitor cocktail). Equivalent protein quantities were subjected to SDS-PAGE, and transferred to PVDF membranes (Millipore). Membranes were blocked with 5% nonfat milk for 1 h at room temperature and then probed with the indicated primary antibodies overnight at 4 °C, followed by the appropriate HRP-conjugated secondary antibodies for 1 h at room temperature. After washing with TBST three times, the blotted membranes were visualized with chemiluminescent kits (Millipore).

### Coimmunoprecipitation assay

HEK293T cells or ESCs were transfected with constructs expressing Flag-Epg5 and HA-Usp8 or HA-Usp8 (C748A) using Lipofectamine 2000 (Invitrogen). Cells were lysed using lysis buffer (50 mM Tris-HCl [pH 7.4], 150 mM NaCl, 1% NP-40, 0.5% sodium deoxycholate, 0.1% SDS and protease inhibitor cocktail) 48 h after transfection. The lysates were immunoprecipitated with 2 μg of specific antibody and 30 -μl A/G agarose beads (Santa Cruz, SC-2003) overnight at 4 °C. The precipitates were washed three times with lysis buffer, and the immune complexes were boiled with loading buffer for 5 min before analysis by SDS-PAGE.

### Protein mass spectrometric analysis

The coiled-coil domain of EPG5 was synthesized as follows: biotin-MNKNEAVSQQIHVLQKEVRQLQAE AAQ (from Beijing SciLight Biotechnology, LCC). One milligram of this short peptide was incubated with ES cells lysates followed by immunoprecipitation with anti-biotin beads. The precipitates were extensively washed with lysis buffer then boiled with SDS loading buffer and subjected to SDS-PAGE. Total proteins were excised from the gels, then subjected to in-gel digestion and analysis by LC-MS/MS.

### Data analysis

Data are expressed as mean ± SEM. Unpaired *t* tests were used to analyze between-group differences. At least three independent experiments were performed in each case.

### Reporting Summary

Further information on experimental design is available in the Nature Research Reporting Summary linked to this article.

## Supplementary information

Supplementary Information

Reporting Summary

## Data Availability

The authors declare that all data supporting the findings of this study are available within the article and its Supplementary Information Files or from the corresponding author upon reasonable request. The mass spectrometry data have been deposited to the Proteome Xchange Consortium via the PRIDE partner repository with the dataset identifier PXD012563.

## References

[1] Klionsky, D. J. Autophagy from phenomenology to molecular understanding in less than a decade. *Nat. Rev. Mol. Cell Biol*. **8**, 931–937 (2007). 10.1038/nrm2245 17712358

[2] Nakatogawa H, Suzuki K, Kamada Y, Ohsumi Y (2009). Dynamics and diversity in autophagy mechanisms: lessons from yeast. Nat. Rev. Mol. Cell Biol..

[3] Feng Y, He D, Yao Z, Klionsky DJ (2014). The machinery of macroautophagy. Cell Res..

[4] He C, Klionsky DJ (2009). Regulation mechanisms and signaling pathways of autophagy. Annu. Rev. Genet..

[5] Mizushima N, Yoshimori T, Ohsumi Y (2011). The role of Atg proteins in autophagosome formation. Annu. Rev. Cell. Dev. Biol..

[6] Levine B, Kroemer G (2008). Autophagy in the pathogenesis of disease. Cell.

[7] Zhang H, Baehrecke EH (2015). Eaten alive: novel insights into autophagy from multicellular model systems. Trends Cell Biol..

[8] Dikic I (2017). Proteasomal and autophagic degradation systems. Annu. Rev. Biochem..

[9] Ho TT (2017). Autophagy maintains the metabolism and function of young and old stem cells. Nature.

[10] Garcia-Prat L (2016). Autophagy maintains stemness by preventing senescence. Nature.

[11] Katajisto P (2015). Stem cells. Asymmetric apportioning of aged mitochondria between daughter cells is required for stemness. Science.

[12] Warr MR (2013). FOXO3A directs a protective autophagy program in haematopoietic stem cells. Nature.

[13] Salemi S, Yousefi S, Constantinescu MA, Fey MF, Simon HU (2012). Autophagy is required for self-renewal and differentiation of adult human stem cells. Cell Res..

[14] Liu P (2017). High autophagic flux guards ESC identity through coordinating autophagy machinery gene program by FOXO1. Cell Death Differ..

[15] Liu K (2016). ATG3-dependent autophagy mediates mitochondrial homeostasis in pluripotency acquirement and maintenance. Autophagy.

[16] Zhu S (2016). p18 inhibits reprogramming through inactivation of Cdk4/6. Sci. Rep..

[17] Wang Z (2016). The Vici syndrome protein EPG5 is a Rab7 effector that determines the fusion specificity of autophagosomes with late endosomes/lysosomes. Mol. Cell.

[18] Zhao H (2013). Mice deficient in Epg5 exhibit selective neuronal vulnerability to degeneration. J. Cell. Biol..

[19] Tian Y (2010). C. elegans screen identifies autophagy genes specific to multicellular organisms. Cell.

[20] Burkhard P, Stetefeld J, Strelkov SV (2001). Coiled coils: a highly versatile protein folding motif. Trends Cell Biol..

[21] Newman JR, Keating AE (2003). Comprehensive identification of human bZIP interactions with coiled-coil arrays. Science.

[22] Herrmann H, Aebi U (2004). Intermediate filaments: molecular structure, assembly mechanism, and integration into functionally distinct intracellular Scaffolds. Annu. Rev. Biochem..

[23] Naviglio S (1998). UBPY: a growth-regulated human ubiquitin isopeptidase. EMBO J..

[24] Mizuno E (2005). Regulation of epidermal growth factor receptor down-regulation by UBPY-mediated deubiquitination at endosomes. Mol. Biol. Cell..

[25] Row PE, Prior IA, McCullough J, Clague MJ, Urbe S (2006). The ubiquitin isopeptidase UBPY regulates endosomal ubiquitin dynamics and is essential for receptor down-regulation. J. Biol. Chem..

[26] Durcan TM (2014). USP8 regulates mitophagy by removing K6-linked ubiquitin conjugates from parkin. EMBO J..

[27] Wang S (2013). Transient activation of autophagy via Sox2-mediated suppression of mTOR is an important early step in reprogramming to pluripotency. Cell. Stem. Cell..

[28] Ma T (2015). Atg5-independent autophagy regulates mitochondrial clearance and is essential for iPSC reprogramming. Nat. Cell Biol..

[29] Wu Y (2015). Autophagy and mTORC1 regulate the stochastic phase of somatic cell reprogramming. Nat. Cell Biol..

[30] Vilchez D (2012). Increased proteasome activity in human embryonic stem cells is regulated by PSMD11. Nature.

[31] Hershko A, Ciechanover A (1998). The ubiquitin system. Annu. Rev. Biochem..

[32] Weidberg H, Shvets E, Elazar Z (2011). Biogenesis and cargo selectivity of autophagosomes. Annu. Rev. Biochem..

[33] Shaid S, Brandts CH, Serve H, Dikic I (2013). Ubiquitination and selective autophagy. Cell Death Differ..

[34] Jin S (2016). USP19 modulates autophagy and antiviral immune responses by deubiquitinating Beclin-1. EMBO J..

[35] Pickart CM (2001). Mechanisms underlying ubiquitination. Annu. Rev. Biochem..

[36] Zhao T (2015). Humanized mice reveal differential immunogenicity of cells derived from autologous induced pluripotent stem cells. Cell. Stem. Cell..

