# Supplementary material for: USP8 maintains embryonic stem cell stemness via deubiquitination of EPG5
Source: Nat Commun. 2019 Apr 1;10:1465. doi: 10.1038/s41467-019-09430-4 (PMC6443784; doi:10.1038/s41467-019-09430-4)
Supplement: Supplementary file 1 — Supplementary Information [file 41467_2019_9430_MOESM1_ESM.pdf]

Supplementary Figures

Supplementary Figure 1 *Epg5* silencing inhibits ESC self-renewal and pluripotency

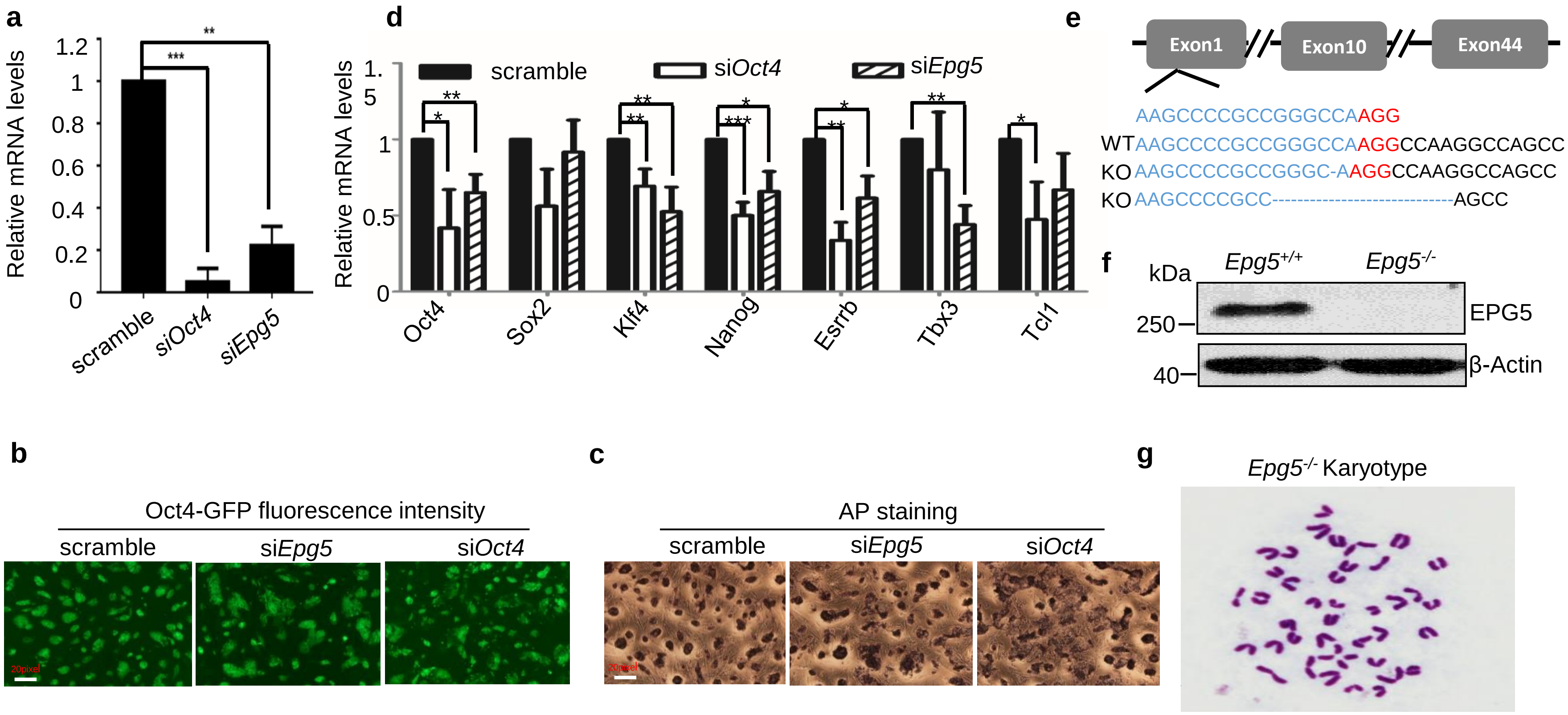

Supplementary Figure 1 Epg5 silencing inhibits ESC self-renewal and pluripotency. **(a)** Transfection of *Oct4*- and *Epg5*-specific siRNAs significantly inhibits *Oct4* and *Epg5* mRNA expression. Error bars indicate the S.D. n = 3. Three independent biological replicates. **, P<0.01, ***, P<0.001 by unpaired two-tailed Student's *t*-test. **(b)** and **(c)** The GFP fluorescence intensity and AP staining of ESC colonies after knockdown of *Oct4* and *Epg5*. Scale bars, 20 pixel **(d)** Knockdown of *Epg5* significantly decreases pluripotency gene expression in ESCs. Error bars indicate the S.D. n = 3. Three independent biological replicates. *, P<0.05; **, P<0.01, ***, P<0.001 by unpaired two-tailed Student's *t*-test. **(e)** The sgRNA designed for targeting the first exon of *Epg5*. **(f)** Western blot analysis of whole cell extracts from $Epg5^{+/+}$ and $Epg5^{-/-}$ ESCs. β-Actin serves as the loading control. **(g)** The $Epg5^{-/-}$ ESCs have a normal karyotype. Scale bars, 2 pm.

Supplementary Figure 2 *Epg5* silencing did not affect ESC apoptosis

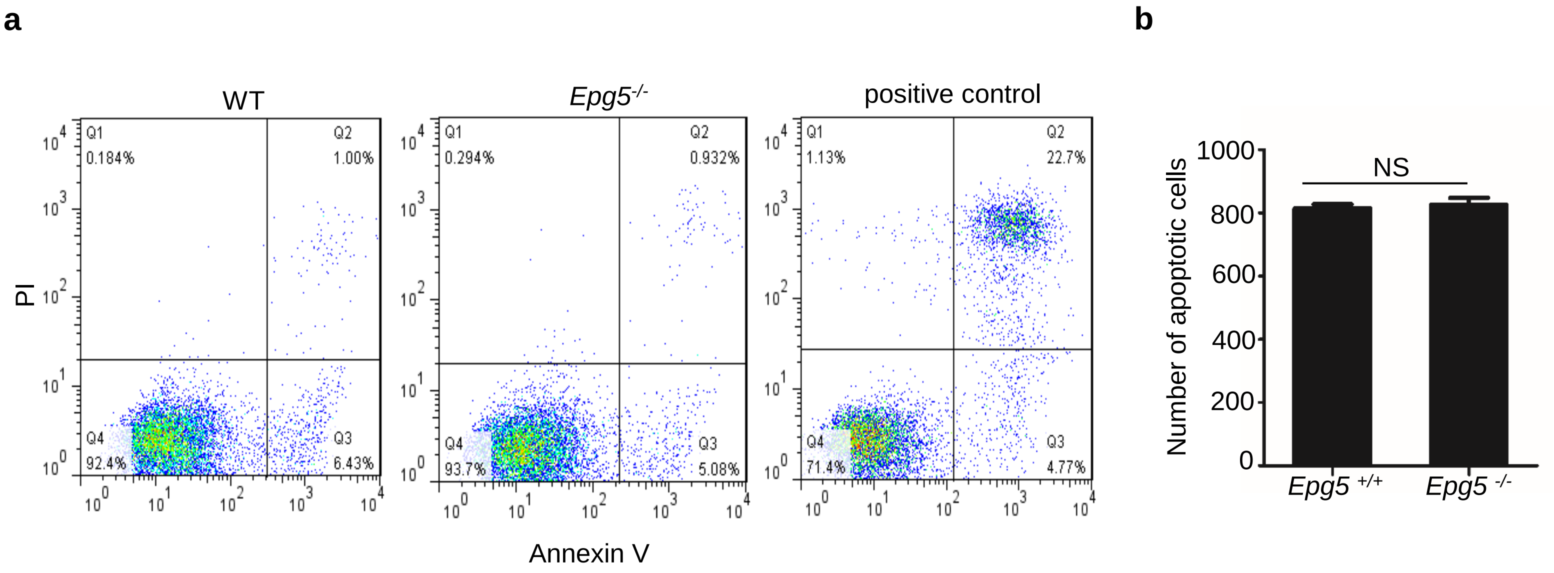

Supplementary Figure 2 *Epg5* silencing does not affect ESC apoptosis. **(a)** and **(b)** The WT, *Epg5*$^{-/-}$ and H_2O_2-treated ESCs were stained with PI and Annexin V and analyzed on a FACS. Double-negative ESCs were counted as viable cells. Cells treated with H_2O_2 were used as a positive control. Error bars indicate the S.D. n = 3. Three independent biological replicates. NS: not significant by unpaired two-tailed Student's *t*-test**.**

Supplementary Figure 3 Identification of USP8 as a regulator to maintain ESC identity

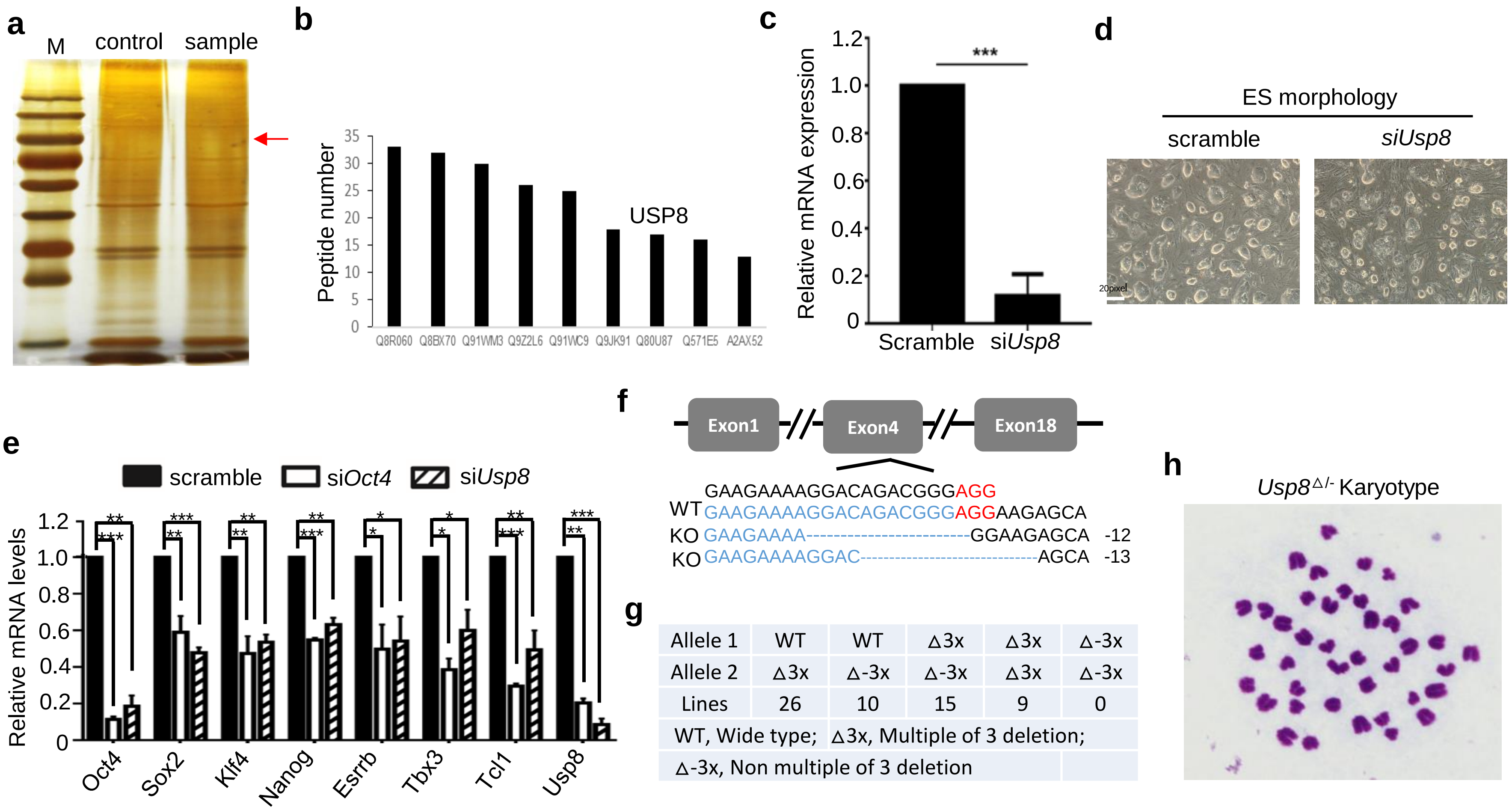

Supplementary Figure 3 Identification of USP8 as a regulator that maintains ESC identity. **(a)** Silver staining of proteins precipitated by the coiled-coil domain of EPG5. The red arrow indicates EPG5 interacting proteins. **(b)** Nine proteins interacting with EPG5 were identified by LC-MS/MS mass spectrometry. **(c)** Transfection of *Usp8*-specific siRNA significantly inhibits *Usp8* mRNA expression. Error bars indicate the S.D. n = 3. Three independent biological replicates. ***, P<0.001 by unpaired two-tailed Student's *t*-test**.** **(d)** The morphology of ESC colonies upon *Usp8* knockdown. Scale bars, 20 pixel. **(e)** The relative mRNA expression of pluripotency genes was detected in WT, si*Oct4* and si*Usp8* ESCs by quantitative PCR. Error bars indicate the S.D. n = 3. Three independent biological replicates. ***, P<0.001, **, P<0.01,*, P<0.05 by unpaired two-tailed Student's *t*-test**.** **(f)** The sgRNA designed for targeting the fourth exon of *Usp8*. Sequencing confirmed an 12 bp deletion in one allele and a 13 bp deletion in another allele (*Usp8*$^{\Delta/-}$), resulting in decreased expression of a truncated USP8. **(g)** Summary of indels obtained on *Usp8* targeting. **(h)** The karyotype of the *Usp8*$^{\Delta/-}$ cell line is normal. Scale bars, 2 pm.

Supplementary Figure 4 *Usp8* decrease did not affect ESC apoptosis

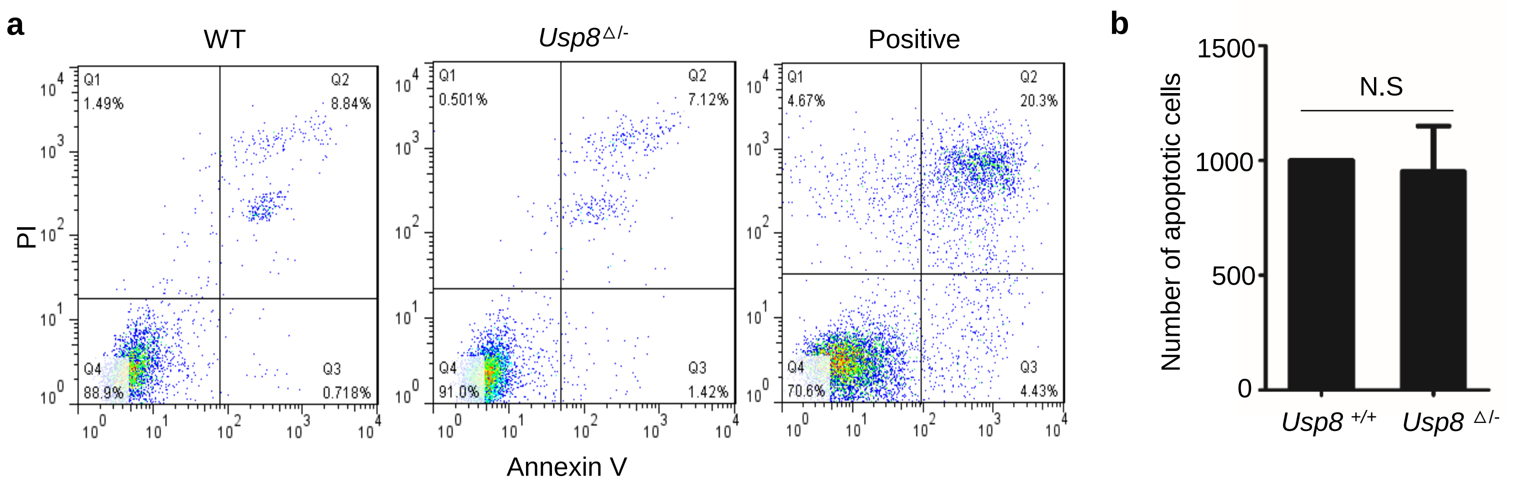

Supplementary Figure 4 *Usp8* reduction does not affect ESC apoptosis. **(a)** and **(b)** The WT, *Usp8*△/- , and H_2O_2-treated ESCs were stained with PI and Annexin V and analyzed on a FACS. Double-negative ESCs were counted as viable cells. Error bars indicate the S.D. n = 3. Three independent biological replicates. NS: not significant by unpaired two-tailed Student's *t*-test**.**

Supplementary Figure 5 EPG5 is degraded by autophagy

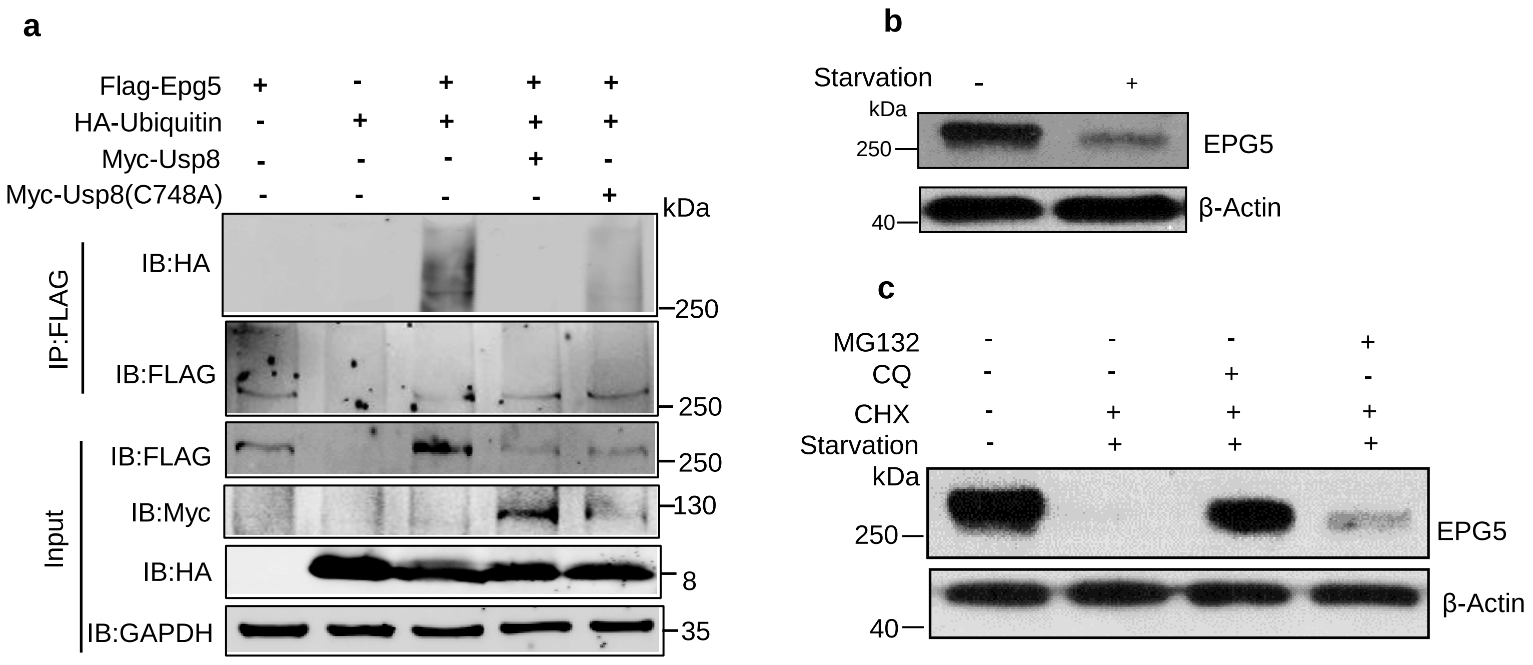

Supplementary Figure 5 EPG5 is degraded by autophagy**. (a)** USP8 deubiquitinates EPG5. Lysates of HEK293T cells transfected with plasmids expressing Flag-*Epg5*, HA-*Ubiquitin*, Myc-*Usp8* or Myc-*Usp8*(C748A) were immunoprecipitated with anti-FLAG and immunoblotted with anti-HA and anti-FLAG. Input cell lysates were immunoblotted with anti-FLAG, anti-Myc, anti-HA and anti-GAPDH as controls. **(b)** Western blot analysis of whole cell extracts from ESCs treated with or without starvation (EBSS starvation for 5h). β-Actin served as the loading control. **(c)** ESCs were treated with or without cycloheximide. Then, western blot analysis of whole cell extracts from ESCs either treated with MG132 or Chloroquine (CQ) under starvation condition. β-Actin served as a loading control.

Supplementary Figure 6 EPG5 and USP8 expression

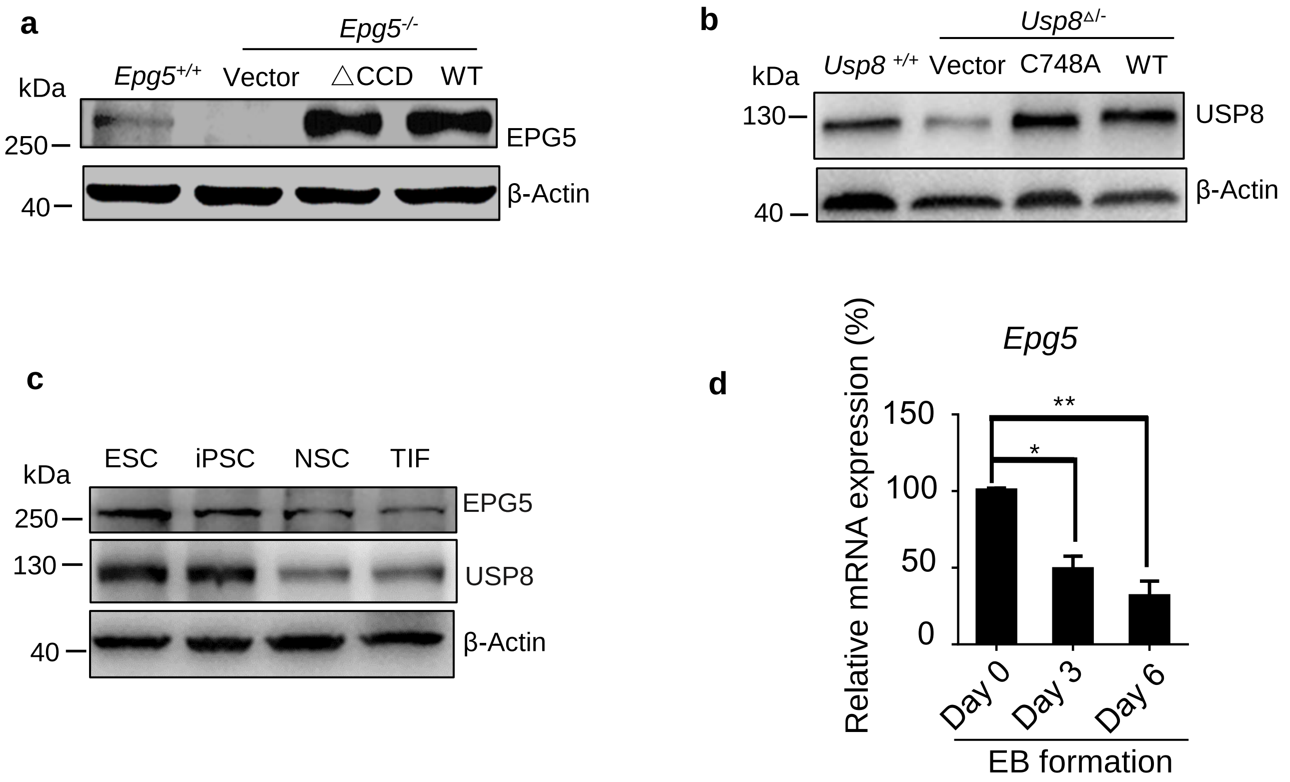

Supplementary Figure 6 EPG5 and USP8 expression. **(a)** Western blotting for EPG5 in WT, *Epg5*$^{-/-}$ ESCs reintroduced of empty vector, WT or ΔCCD mutant *Epg5*. β-Actin served as a loading control. **(b)** Western blotting for *Usp8*$^{+/+}$ ESCs and *Usp8*$^{\Delta/-}$ ESCs reintroduced of empty vector, WT or C748A mutant *Usp*8. **(c)** Western blotting for EPG5 and USP8 in mouse ESC, iPSC, Neuron stem cell (NSC) and tail fibroblast (TIF). Data shown are representative of 3 independent experiments. **(d)** *Epg5* expression decreased along with embryoid body (EB) differentiation. The relative mRNA expression levels of *Epg5* during EB differentiation by quantitative PCR on the indicated days. Error bars indicate the S.D. n = 3. Three independent biological replicates. **, P<0.01, *, P<0.05 by unpaired two-tailed Student's t-test.

Supplementary Figure 7 USP8 regulates ESC self-renewal and pluripotency through deubiquination of EPG5

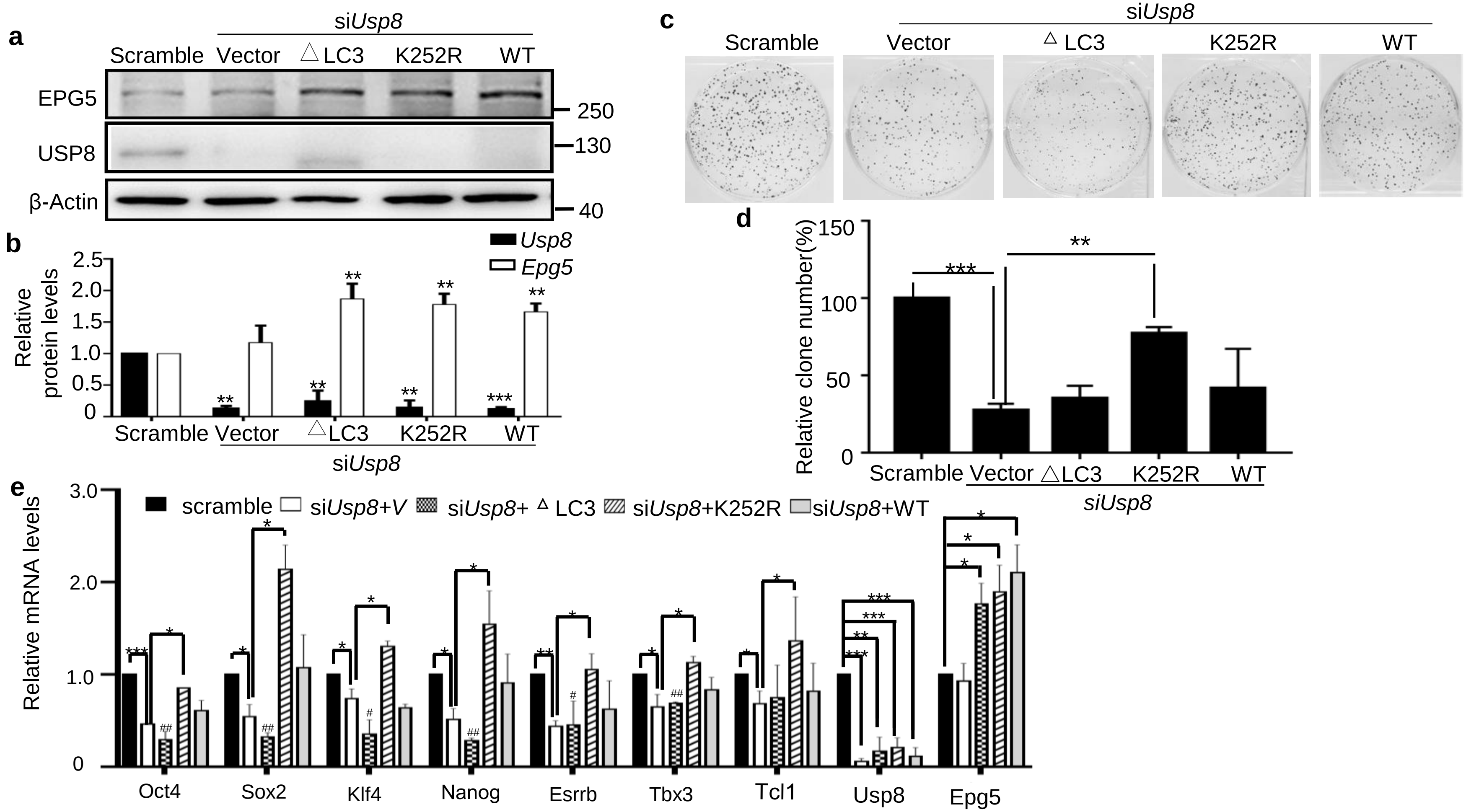

Supplementary Figure 7 USP8 regulates ESC self-renewal and pluripotency through deubiquination of EPG5. **(a)** Western blot analysis of whole cell extracts from scramble, si*Usp8*+vecor*,* si*Usp8*+ △-LC3*,* si*Usp8*+K252R and si*Usp8*+WT ESCs. β-Actin served as a loading control. **(b)** Quantification of the EPG5 and USP8 expression in **a**. Error bars indicate the S.D. n = 3. Three independent biological replicates. **, P<0.01, ***, P<0.001 by unpaired two-tailed Student's *t*-test**. (c)** The reduced colony formation ability of si*Usp8* ESCs is rescued by introduction of K252R *Usp8* but not the wide-type or △-LC3 *Usp8* mutant. **(d)** Quantification of the colony formation in **C**. Error bars indicate the S.D. n = 3. Three independent biological replicates. **, P<0.01, ***, P<0.001 by unpaired two-tailed Student's *t*-test**. (e)** The relative mRNA expression of pluripotency genes in scramble, si*Usp8*+vecor*,* si*Usp8*+ △-LC3*,* si*Usp8*+K252R and si*Usp8*+WT ESCs was detected by quantitative PCR. Error bars indicate the S.D. n = 3. Three independent biological replicates. #, siUsp8+△ LC3 compare to scramble, *, #<0.05, **, #<0.01, ***, #<0.001**, P<0.01, ***, P<0.001 by unpaired two-tailed Student's *t*-test**.**

Supplementary Figure 8 Depletion of EPG5 significantly decreased autophagic flux in ESCs

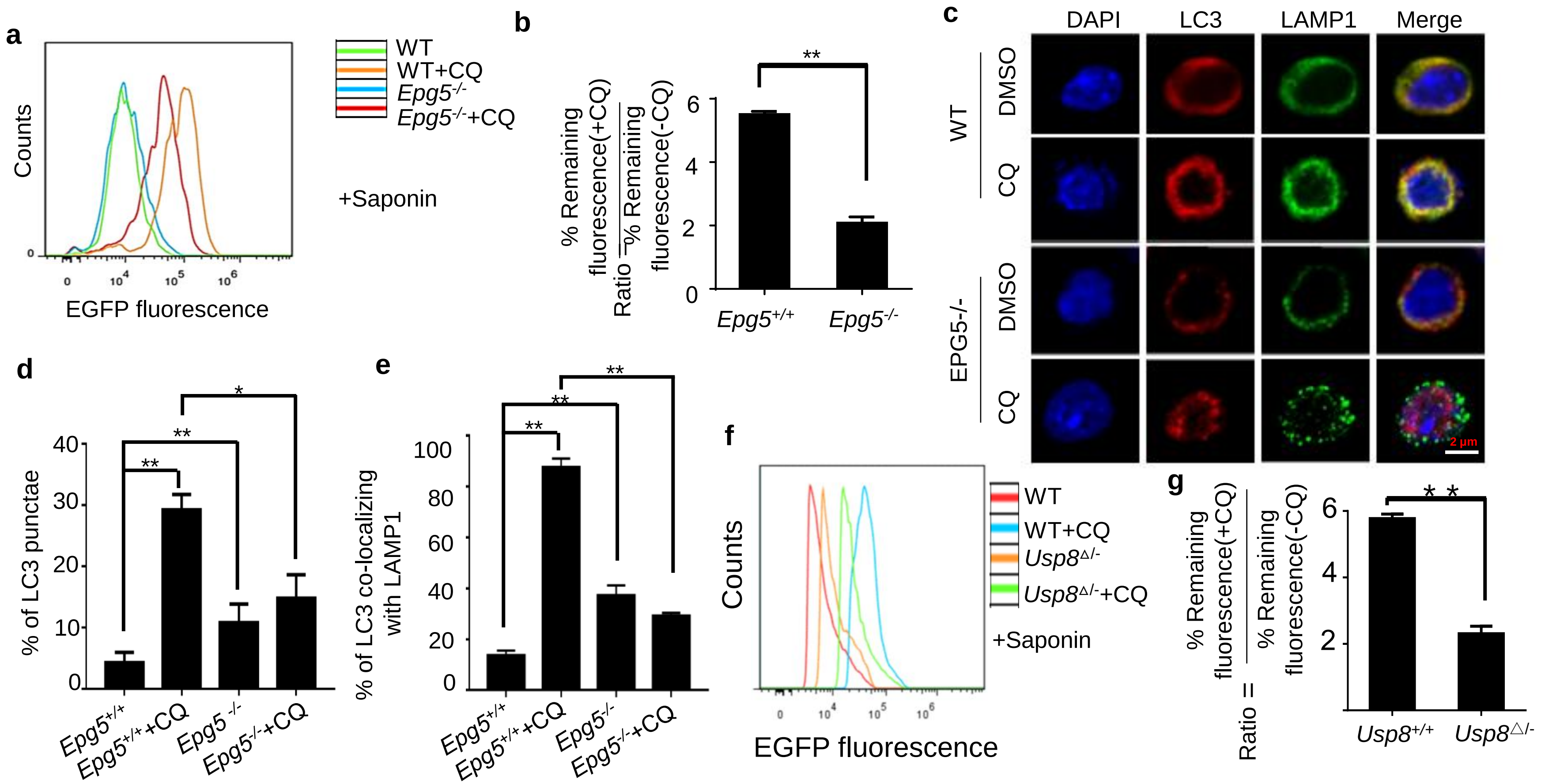

Supplemental figure 8 Depletion of EPG5 significantly decreased autophagic flux in ESCs. **(a)** and **(b)** Flow cytometry for EGFP-LC3 in *Epg5*$^{+/+}$ and *Epg5*$^{-/-}$ ESCs treated with or without 10 nM chloroquine (CQ) for 5h. Error bars indicate the S.D. n = 3. Three independent biological replicates. **, P<0.01 by unpaired two-tailed Student's *t*-test. **(c)** Confocal fluorescence micrographs of *Epg5*$^{+/+}$ and *Epg5*$^{-/-}$ ESCs treated with DMSO or 10 nM CQ for 5 hours. Images showing LAMP1 (green) and LC3 (red) co-localization and LC3 punctae. DAPI(blue), Scale bars, 2 μm. **(d)** Quantification of LC3 punctae in *Epg5*$^{+/+}$ and *Epg5*$^{-/-}$ ESCs in the presence or absence of CQ. Error bars indicate the S.D. n = 3. Three independent biological replicates. *, P<0.05, **, P<0.01 by unpaired two-tailed Student's *t*-test. **(e)** Quantification of LAMP1 and LC3 colocalization in *Epg5*$^{+/+}$ and *Epg5*$^{-/-}$ ESCs in the presence or absence of CQ. Error bars indicate the S.D. n = 3. Three independent biological replicates. **, P<0.01 by unpaired two-tailed Student's *t*-test. **(f)** and **(g)** Flow cytometry for EGFP-LC3 in *Usp8*$^{+/+}$ and *Usp8*$^{\Delta/-}$ ESCs treated with or without 10 nM chloroquine (CQ) for 5h. Error bars indicate the S.D. n = 3. Three independent biological replicates. **, P<0.01 by unpaired two-tailed Student's *t*-test.

Supplementary Figure 9 USP8 and EGP5 decreases did not induce expression of differentiation marker

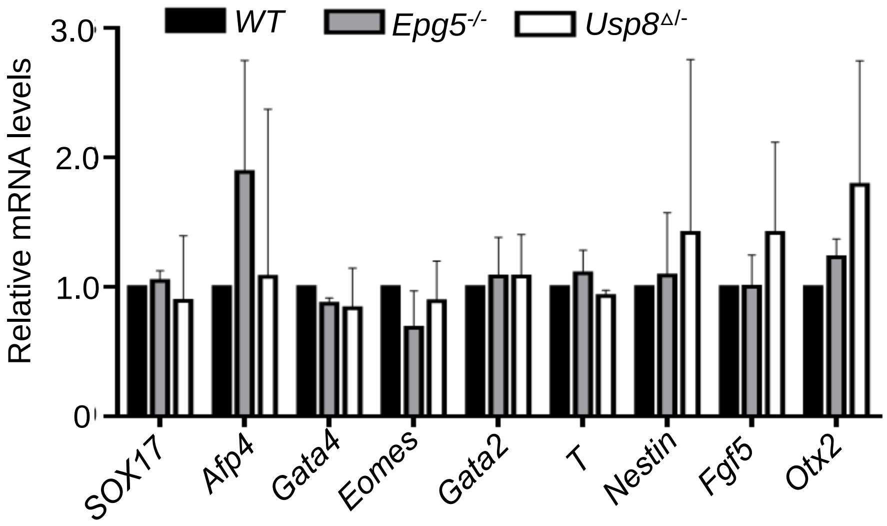

Supplemental figure 9 USP8 and EGP5 decreases did not induce differentiation marker expression. The relative mRNA expression of 3 germ layer genes in *WT, Epg5$^{-/-}$ and Usp8$^{\Delta/-}$* ESCs. Error bars indicate the S.D. n = 3. Three independent biological replicates. NS, not significant by unpaired two-tailed Student's *t*-test**.**

Supplementary Figure 10 EPG5 or USP8 silencing inhibits hESC self-renewal and pluripotency

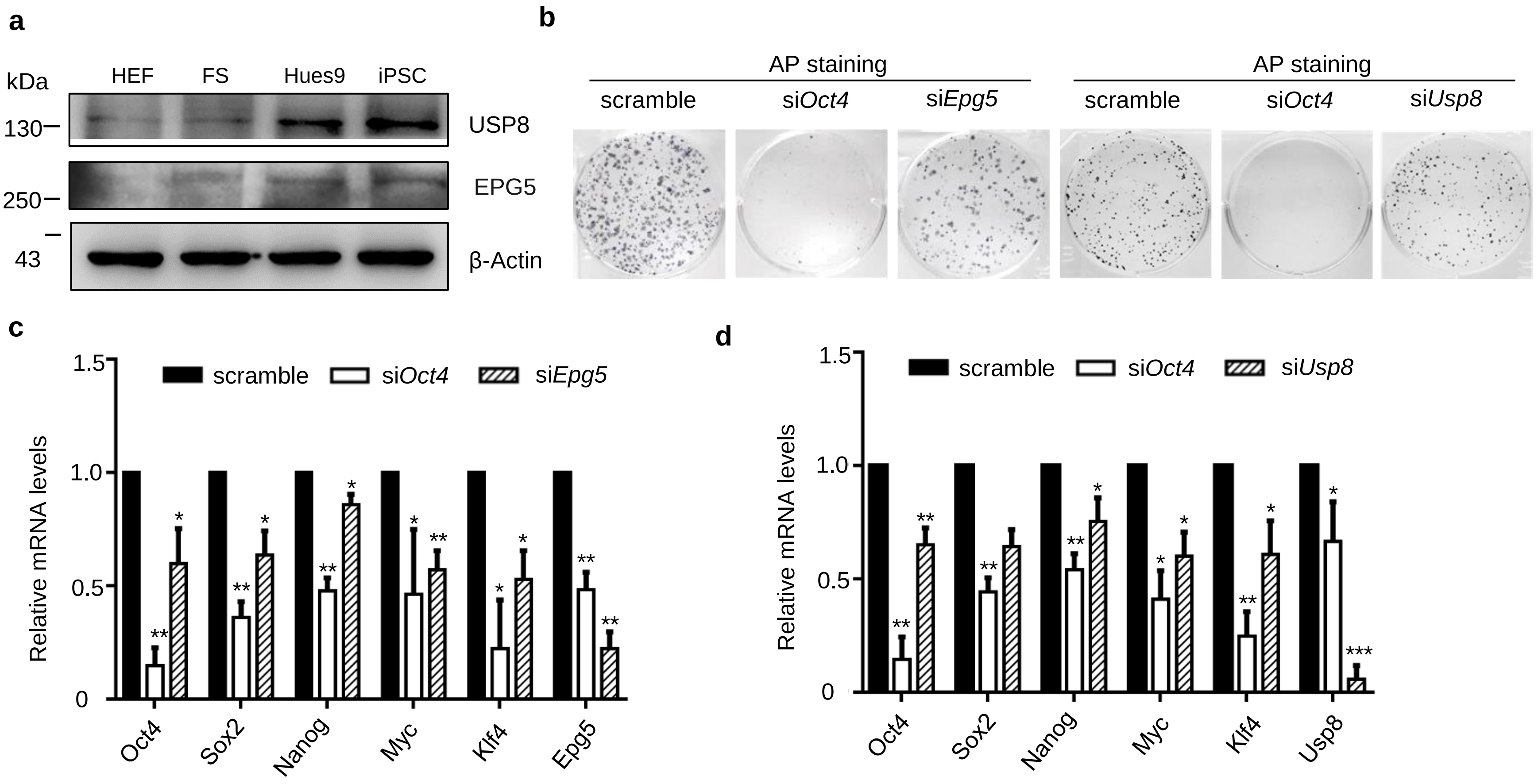

Supplementary Figure 10 *Epg5* or *Usp8* silencing inhibits human ESC self-renewal and pluripotency. **(a)** Western blotting for USP8, EPG5 and β-actin in human embryonic fibroblast (HEF), Foreskin (FS), Hues9 ESC and iPSC. Images representative of 3 independent experiments. **(b)** Knockdown of *Epg5* or *Usp8* by specific siRNAs inhibits self-renewal of human ESCs.. **(c)** and **(d)** The relative mRNA expression of pluripotency genes was detected in hESCs transfected with scramble, si*Oct4* and si*Epg5* siRNA. Error bars indicate the S.D. n = 3. Three independent biological replicates. ***, P<0.001, **, P<0.01,*, P<0.05 by unpaired two-tailed Student's *t*-test (si*Usp8* or si*Epg5*, si*Oct4* compare to scramble).

Supplementary Figure 11 Original source data of the blotting images

Figure 1b

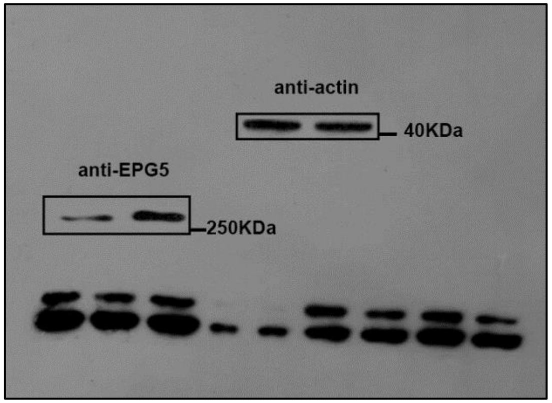

Figure 2a

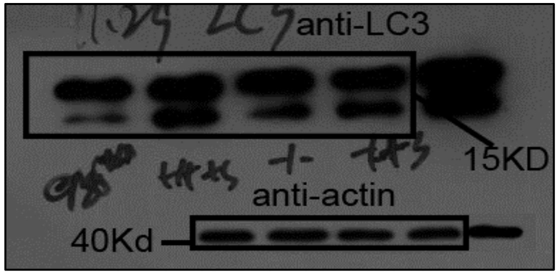

Figure 2c

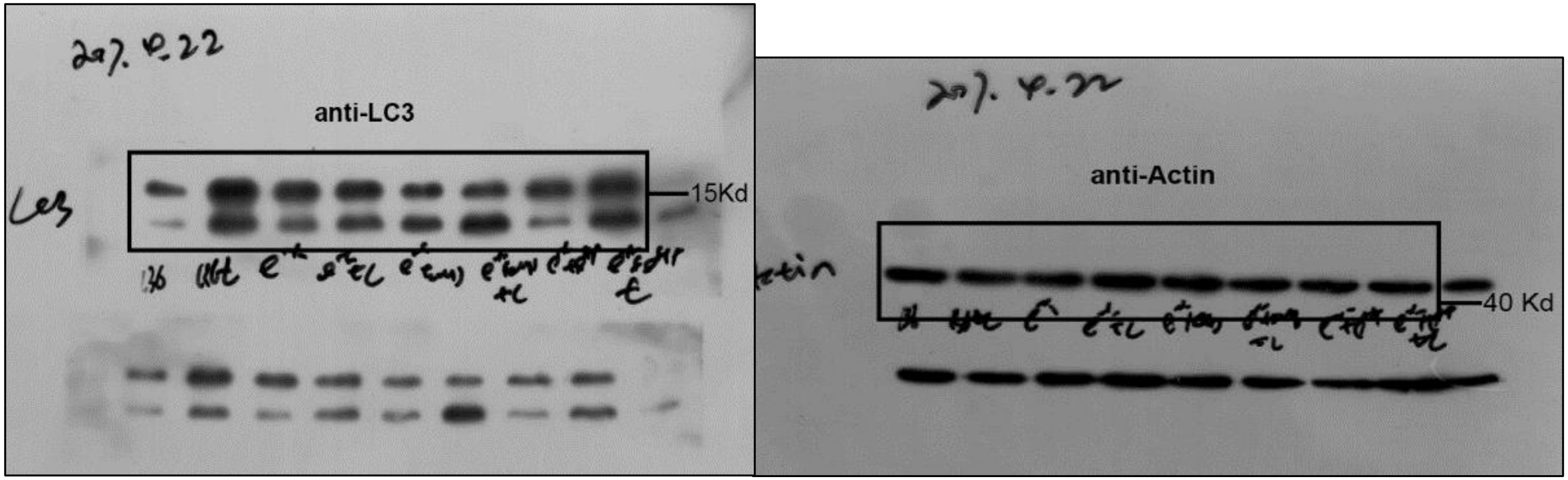

Figure 3b

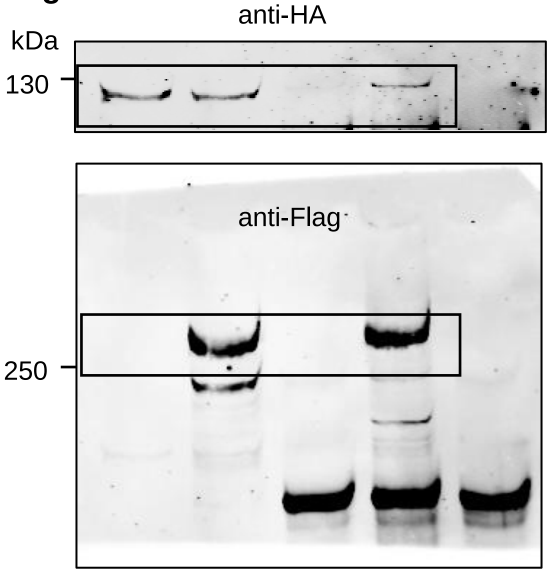

Figure 3c

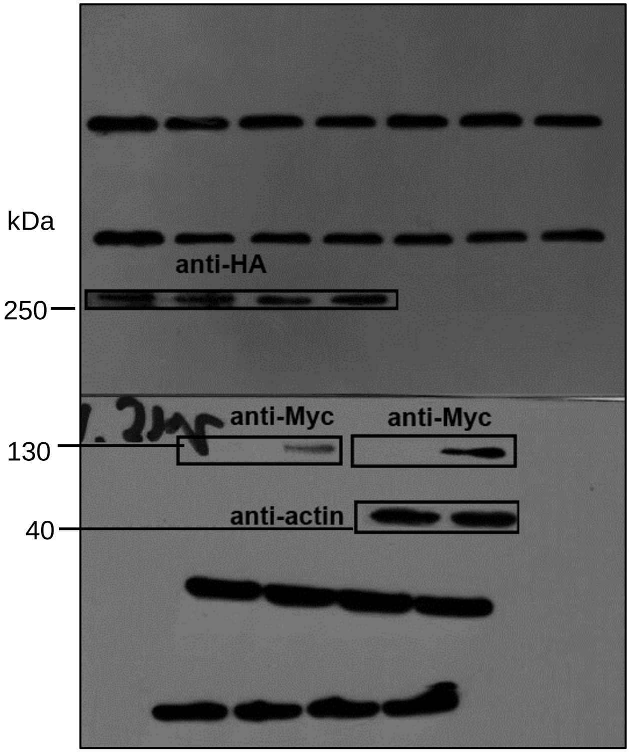

Figure 3d

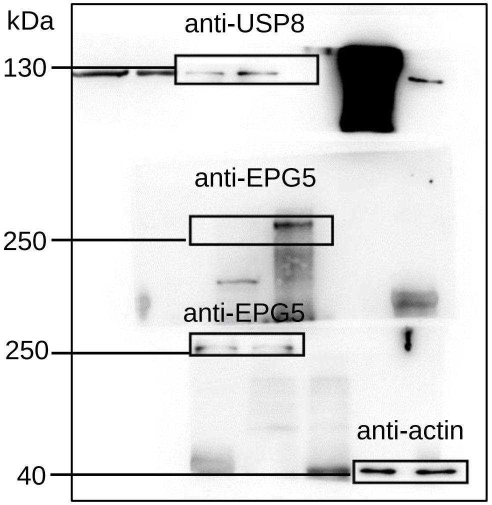

Figure 4a

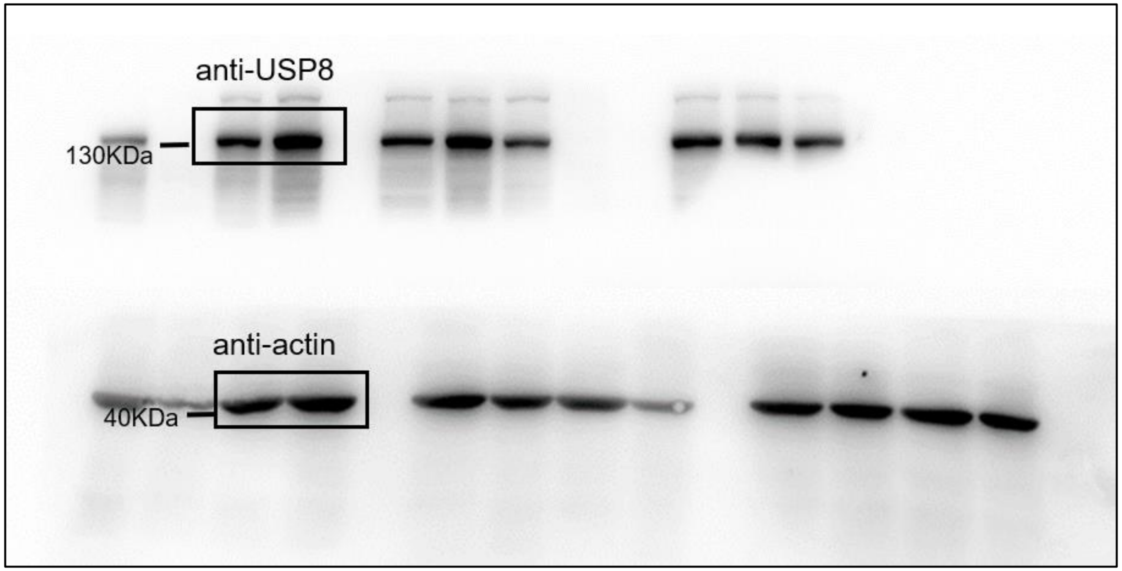

Figure 5a

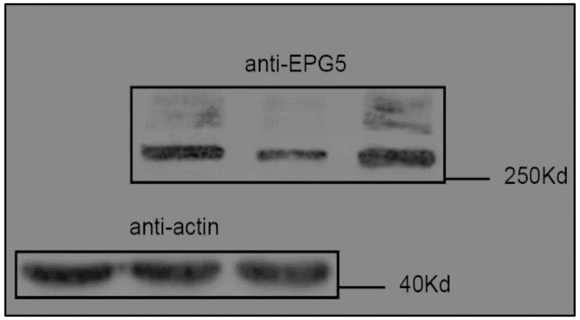

Figure 5b

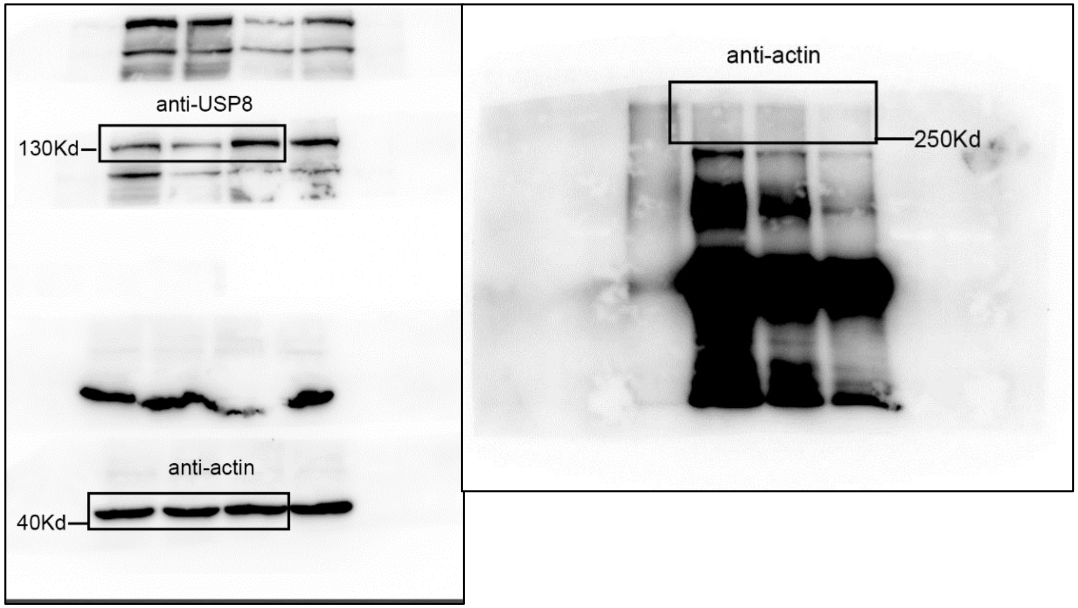

Figure 5c

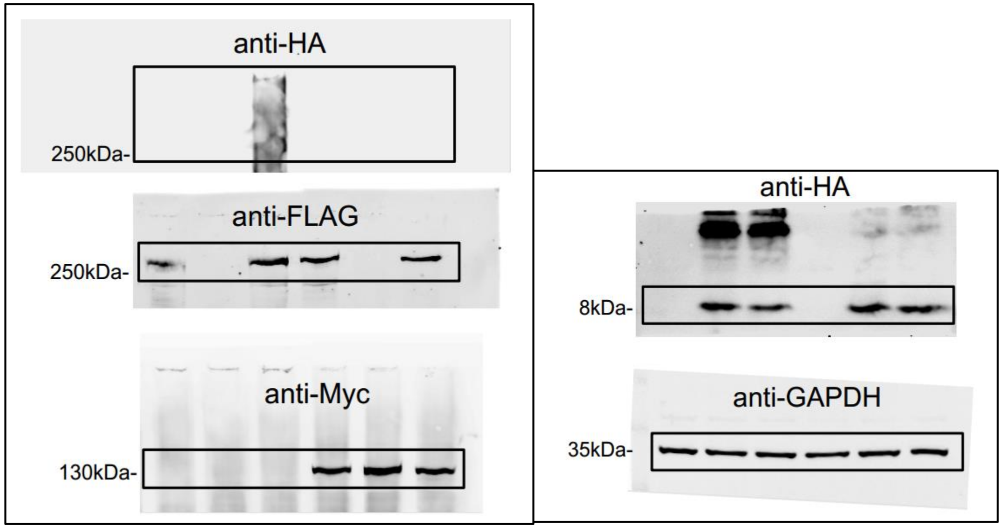

Figure 5d

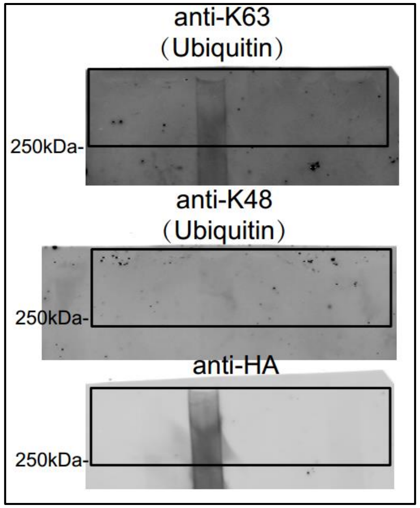

Figure 5e

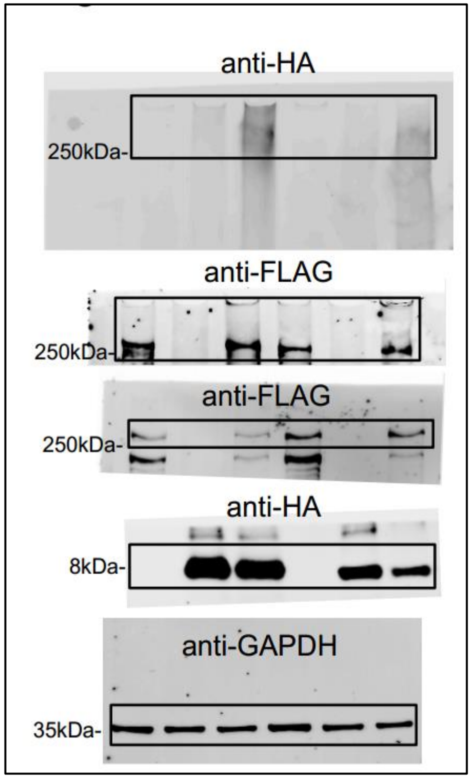

Figure 5g

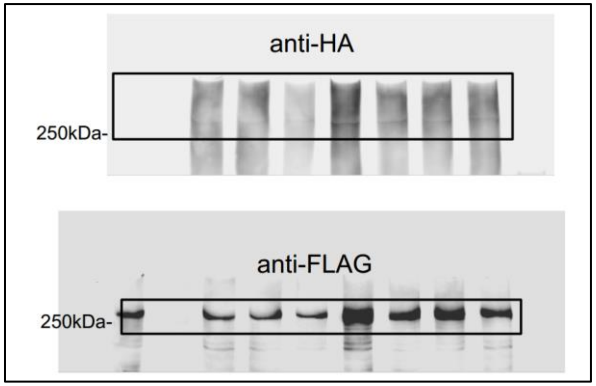

Figure 6a

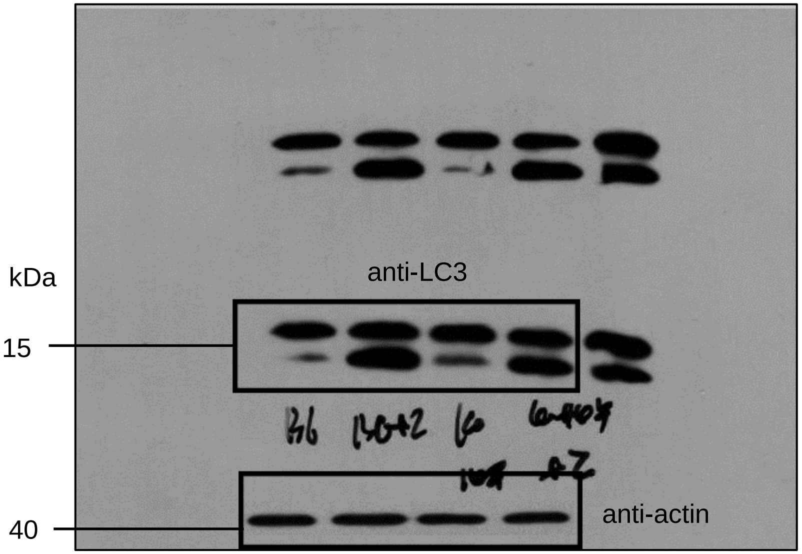

Figure 6c

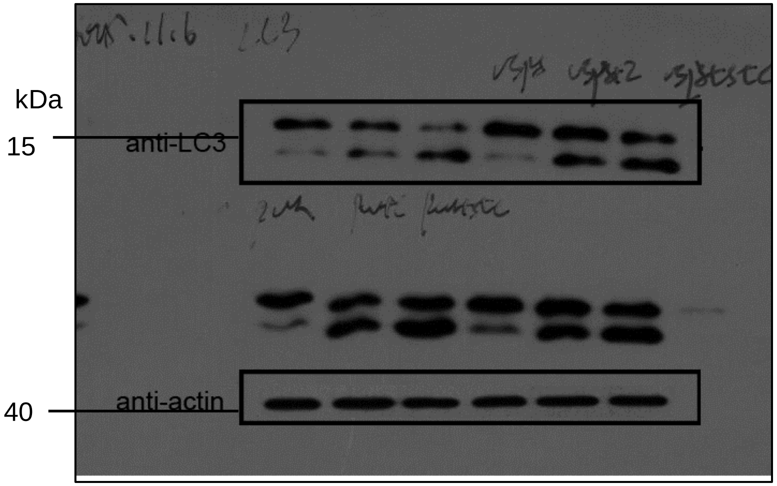

Figure 6f

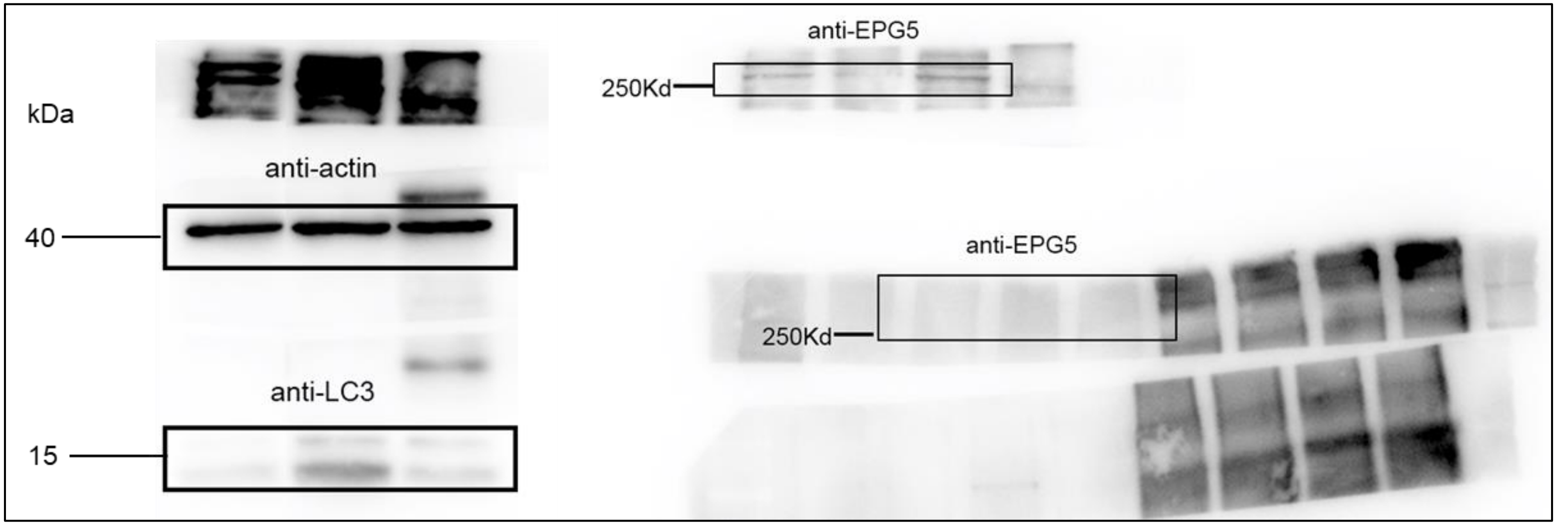

Supplementary Figure 1f

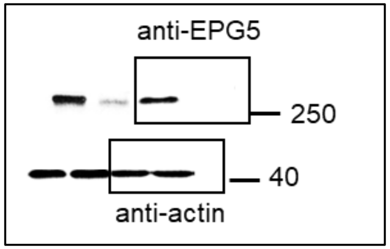

Supplementary Figure 5a

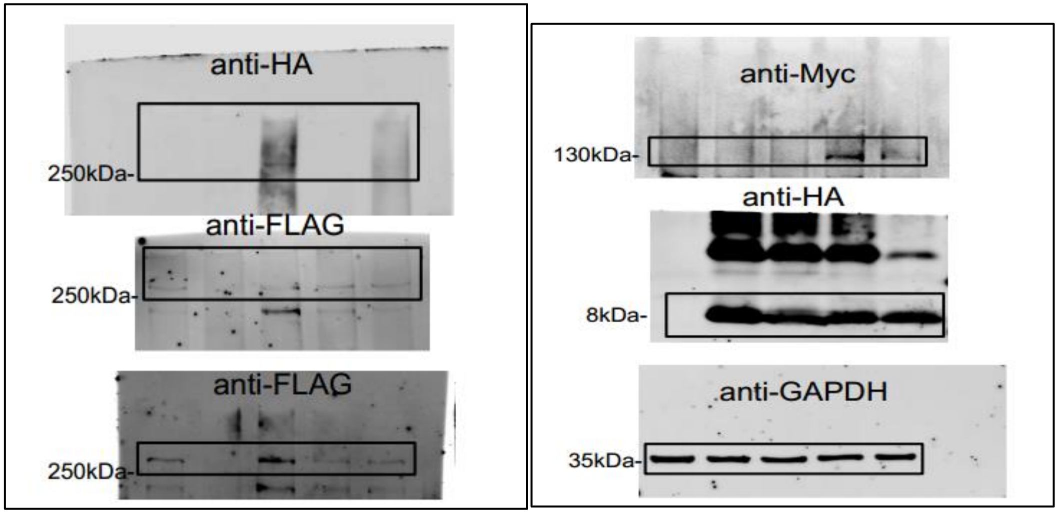

Supplementary Figure 5b

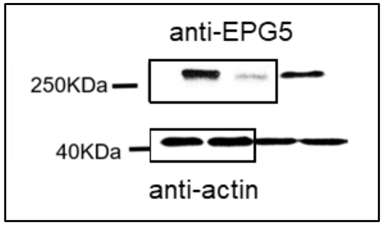

Supplementary Figure 5c

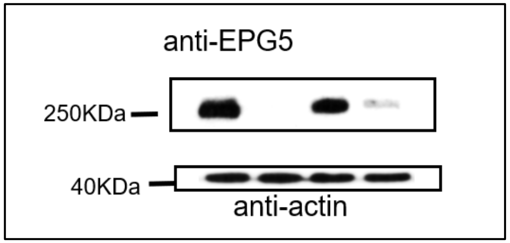

Supplementary Figure 6a

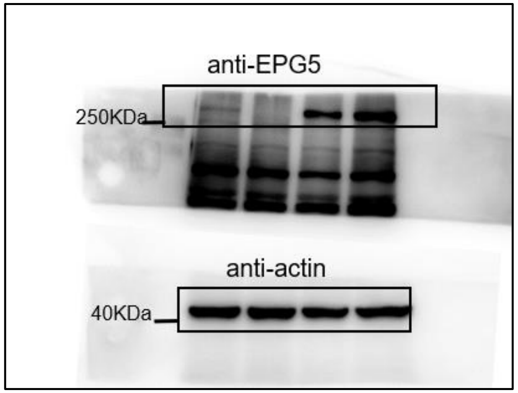

Supplementary Figure 6b

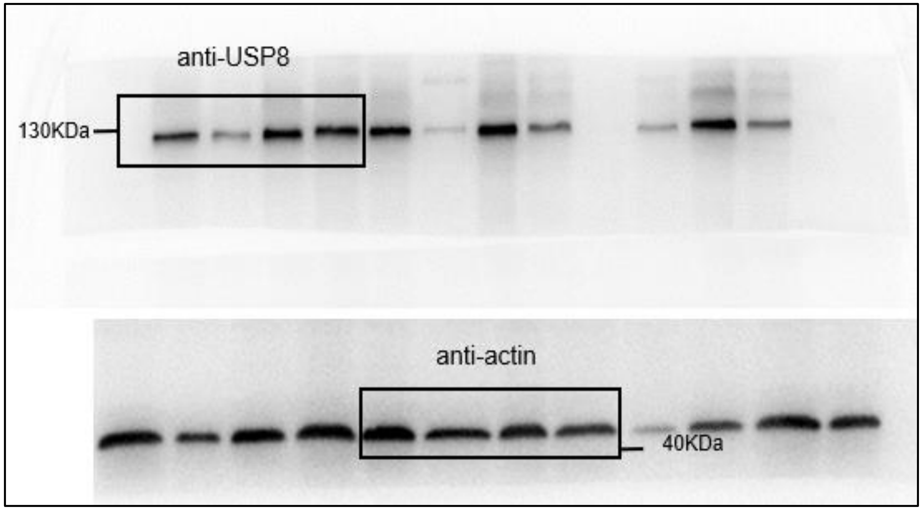

Supplementary Figure 6c

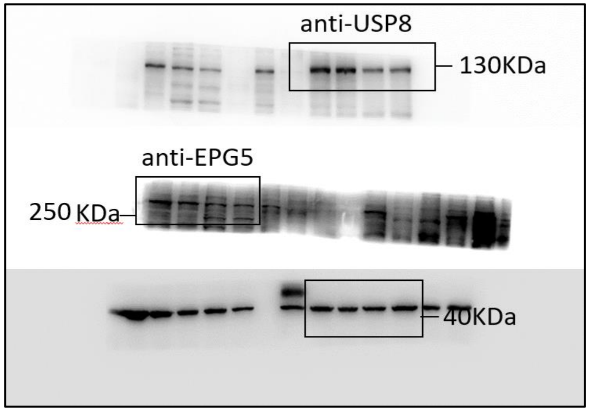

Supplementary Figure7a

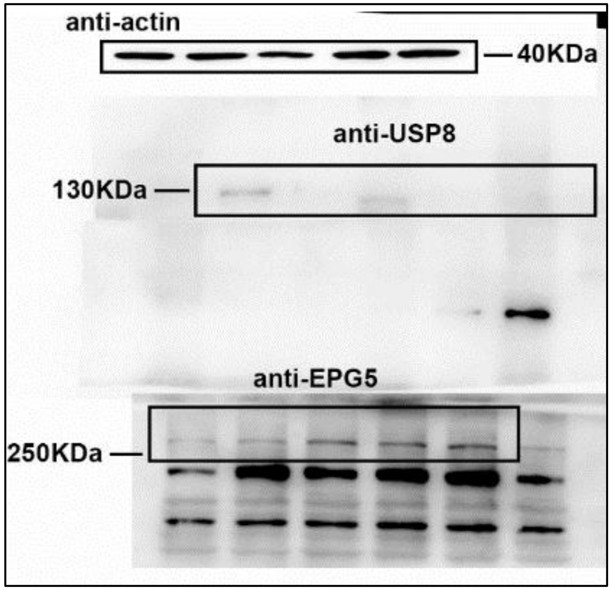

Supplementary Figure10a

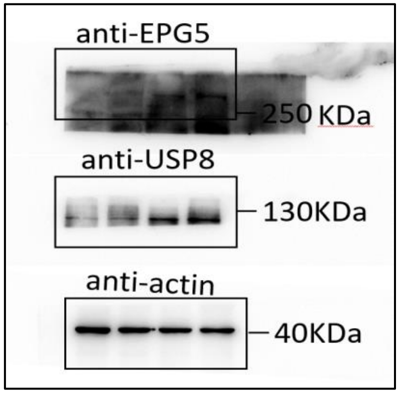

Supplementary Tables

Supplementary Table 1

Primer name	Species	Forward sequences (5′ - 3′)	Reverse sequences (5′ - 3′)
EPG5	Mouse	TTGGCTTTGTAACTGGTCGG	CACAGGCAGCCTTCTTTGTTC
USP8	Mouse	AGACTCTCCGAAAGCCTTAAACT	GCCGTTAATCCTTTGGGTTTTGG
Oct4	Mouse	GTGGAGGAAGCCGACAACAATGA	CAAGCTGATTGGCGATGTGAG
Sox2	Mouse	TTGCAAAGGGTTTTCGAGAC	TGGAGGACTCATCCGAAGTC
Nanog	Mouse	AAATTCCAGGTGATCTTGCG	TGTCCTTGGGGTACAGTTGC
Klf4	Mouse	GTGCCCC GACTAACCGTTG	GTCGTTGAACTCCTCGGTCT
Nanog	Mouse	TCTTCCTGGTCCCCACAGTTT	GCAAGAATAGTTCTCGGGATGAA
Esrrb	Mouse	CAGGCAAGGATGACAGACG	GAGACAGCACGAAGGACTGC
Sox17	Mouse	GATGCGGGATACGCCAGTG	CCACCACCTCGCCTTTCAC
Gata4	Mouse	CCCTACCCAGCCTACATGG	ACATATCGAGATTGGGGTGTCT
Gata2	Mouse	CACCCCGCCGTATTGAATG	CCTGCGAGTCGAGATGGTTG
Nestin	Mouse	CCCTGAAGTCGAGGAGCTG	CTGCTGCACCTCTAAGCGA
Otx2	Mouse	TATCTAAAGCAACCGCCTTACG	AAGTCCATACCCGAAGTGGTC
Fgf5	Mouse	CTGTATGGACCCACAGGGAGTAAC	ATTAAGCTCCTGGGTCGCAAG
T	Mouse	GCTTCAAGGAGCTAACTAACGAG	CCAGCAAGAAAGAGTACATGGC
Eomes	Mouse	GCGCATGTTTCCTTTCTTGAG	GGTCGGCCAGAACCACTTC
Afp4	Mouse	CTTCCCTCATCCTCCTGCTAC	ACAAACTGGGTAAAGGTGATGG
β-actin	Mouse	GGCTGTATTCCCCTCCATCG	CCAGTTGGTAACAATGCCATGT
EPG5	Human	GCCATTGCCTTCAAGATGGG	TGGCCAAACTGAGCTCTACA
USP8	Human	AAGGAGCAATCACAGCAAAGG	CTGCATTCTTCGAGCATCCATTA
Oct4	Human	TTCGGGCACTGCAGGAACAAATTC	TATGCAAAGCAGAAACCCTCGTGC
Sox2	Human	CACAGCAAATGACAGCTGCAA	GTCGGCATCGCGGTTTT
Nanog	Human	TTTGTGGGCCTGAAGAAAACT	AGGGCTGTCCTGAATAAGCAG
Myc	Human	ACTCTGAGGAGGAACAAGAA	TGGAGACGTGGCACCTCTT
Kl4	Human	TCTCAAGGCACACCTGCGAA	TAGTGCCTGGTCAGTTCATC
β-actin	Human	TGAAGTGTGACGTGGACATC	GGAGGAGCAATGATCTTGAT

Supplementary Table 1 List of primers for Quantitative real-time PCR
